# Supplementary material for: Task-relevant representational spaces in human memory traces
Source: PLoS Comput Biol. 2025 Sep 12;21(9):e1013476. doi: 10.1371/journal.pcbi.1013476 (PMC12448971; doi:10.1371/journal.pcbi.1013476)
Supplement: S1 Text — (PDF) [file pcbi.1013476.s001.pdf]

Supplementary Materials (S1 Text)

Task-relevant representational spaces in human memory traces

Rebekka Heinen[1*&], Elias M.B. Rau[1&] , Nora A. Herweg[1&], Nikolai Axmacher[1]

1 Department of Neuropsychology, Institute of Cognitive Neuroscience, Faculty of Psychology, Ruhr University Bochum, Bochum, Germany.

& Equal contributions

* rebekka.heinen@ruhr-uni-bochum.de

Fig A
Mixed blocks reflect perceptual and conceptual formats on single dimension levels

Fig B
Overview of participant ratings for study I+II and relation to DNN similarities.

Fig C
Layer-wise and averaged representational distances in Deep Neural Networks reflect behavioral similarity judgements

Fig D
Reaction times are influenced by rated distance

Fig E
Representational distance affects subsequent memory regardless of perceptual/conceptual encoding space and cueing

Fig F
Layer-wise correspondence of representational distances in DNNs to binary ratings study III

Fig G
Effects of DNN-based representational similarities on memory strength during the general and the specific memory task

Fig H
Different formats contribute to memory strength and correct rejection performance depending on task instructions at retrieval

Fig I
Conceptual cueing specifically influences semantic memory

Table A
Linear mixed models and their beta weights from main results

Table B
Linear mixed models and their beta weights from Supplementary analyses

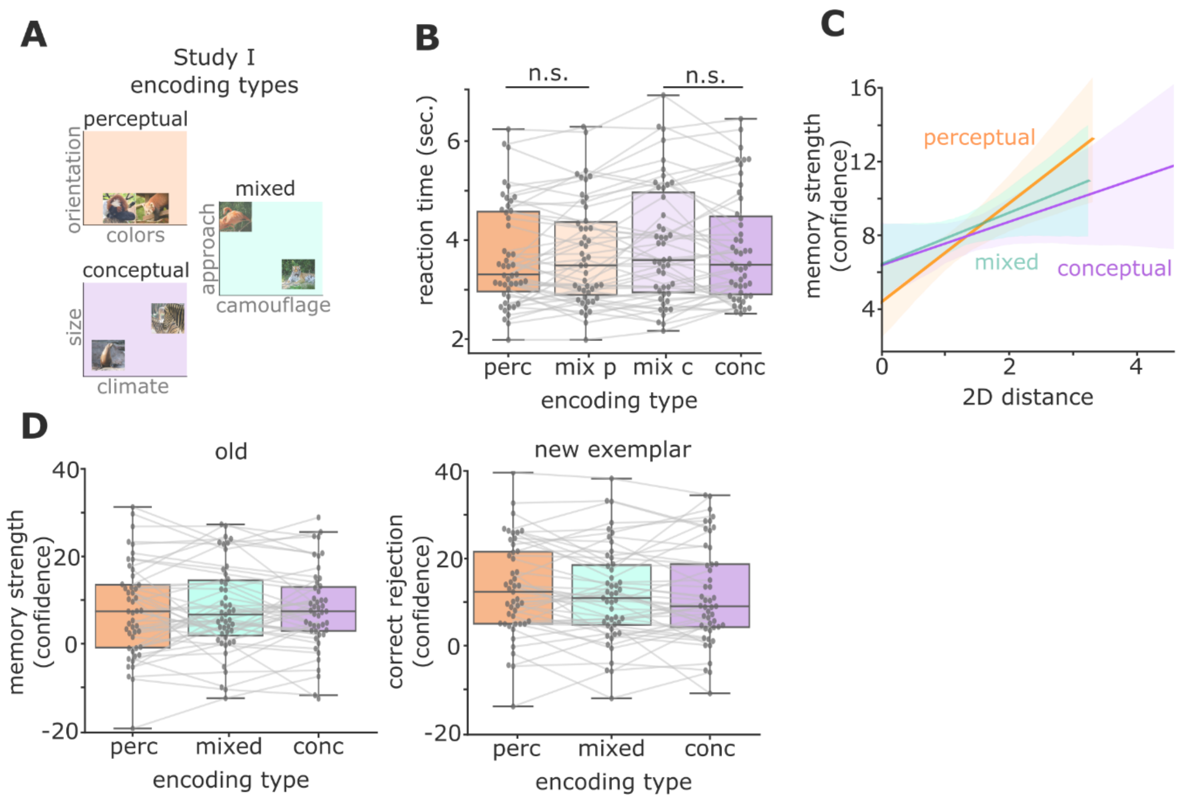

Fig A: Mixed blocks reflect perceptual and conceptual formats on single dimension levels: (A) In Study I, we presented perceptual blocks (two perceptual dimensions), conceptual blocks (two conceptual dimensions), and mixed blocks (one perceptual and one conceptual dimension). We investigated whether ratings in mixed blocks would differ from ratings in congruent (i.e., purely perceptual, and purely conceptual) blocks. (B) We used linear mixed model to predict reaction times using encoding type (perceptual/conceptual/mixed) as a predictor, including a random participant intercept. Reaction times during perceptual ratings in mixed blocks ($z = -1.65$, $\chi^2_{(1)} = 2.72$, $p = 0.098$; likelihood ratio test) and during conceptual ratings in mixed blocks ($z = 1.38$, $\chi^2_{(1)} = 1.91$, $p = 0.167$; likelihood ratio test) did not differ from their congruent counterpart. (C) We then assessed whether the influence of rated distances on memory strength differs between encoding tasks. Using a linear mixed model including the interaction of encoding task (conceptual, perceptual, mixed) and 2D distance as predictors, we found no interaction of 2D distances and encoding task (perceptual vs conceptual: $z = 1.05$, mixed vs conceptual: $z = 1.04$, mixed vs perceptual: $z = 1.38$, $\chi^2_{(2)} = 2.89$, $p = 0.235$; likelihood ratio test). (D) Memory strength (left; perceptual versus conceptual: $z = 0.49$, mixed vs conceptual: $z = -0.11$, mixed vs perceptual: $z = 0.64$, $\chi^2_{(1)} = 0.42$, $p = 0.810$; likelihood ratio test) and correct rejection of new exemplars (right; conceptual vs perceptual: $z = -1.74$, mixed vs perceptual: $z = -1.61$, mixed vs conceptual: $z = -0.10$, $\chi^2_{(1)} = 3.78$, $p = 0.151$; likelihood ratio test) did not differ between blocks. Based on these results, we decided to concatenate perceptual dimension ratings from mixed and perceptual blocks and conceptual ratings from mixed and conceptual blocks for further analysis of DNN-based similarities in Study I. Data are visualized after removing participant-wise estimated random effects. 95% confidence intervals, center line indicates median, box limits indicate upper and lower quartiles, whiskers indicate 1.5 interquartile range, individual points represent participant averages. n.s., not significant

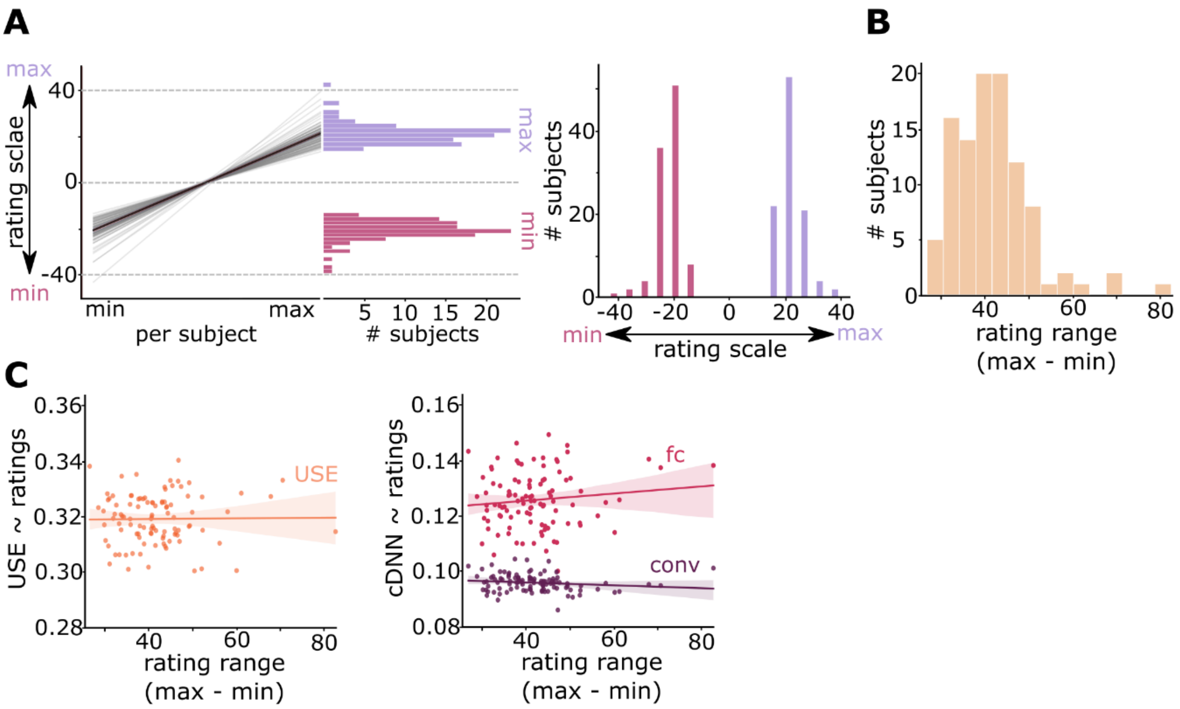

Fig B: Overview of participant ratings for Study I+II and relation to DNN similarities. (A) Participants in Study I and II indicated similarity judgements on a visual analogue scale. We plotted histograms of minimum and maximum ratings per participant, suggesting only minor variations in the usage of the rating scale across participants (95% of participants within a range of -30 to 30). (B) Histogram of rating range (maximum rating – minimum rating) for all participants. (C) Finally, to test if the individual rating range would influence the correlation of participant ratings and DNN similarity matrices, we correlated the average DNN similarity for conv, fc and USE in each participant to the participants' rating range (max – min) using Pearson correlation. We show that all three layer/model types do not relate to participant rating ranges (USE: r = 0.012, p = 0.90; fc: r = 0.12, p = 0.24; conv: r = -0.15, p = 0.13).

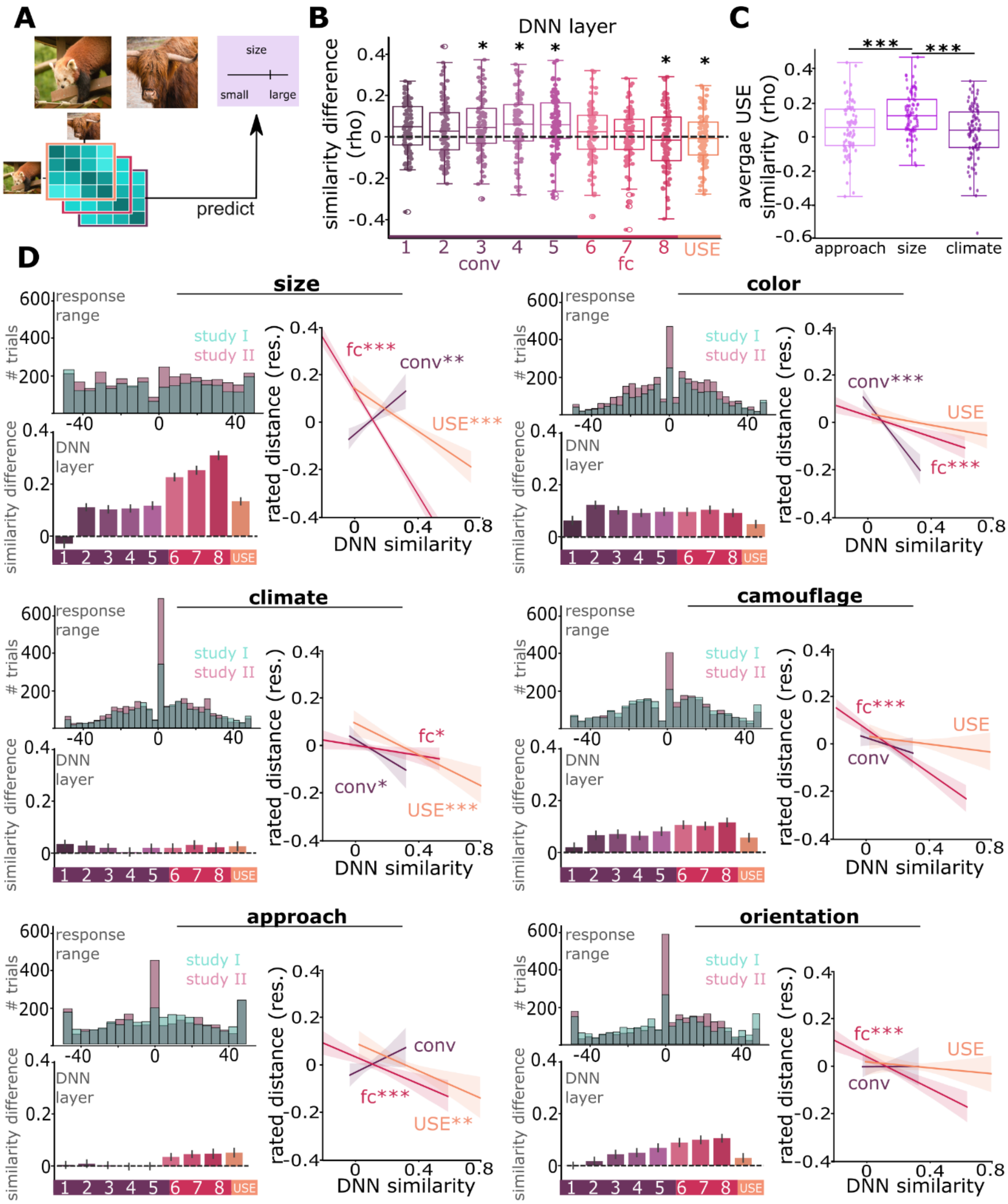
A
size
small
large
predict
B
DNN layer
similarity difference (rho)
conv
fc
USE
C
avergae USE similarity (rho)
approach
size
climate
D
size
color
climate
camouflage
approach
orientation
response range
trials
study I
study II
DNN layer
similarity difference
rated distance (res.)
DNN similarity
fc***
conv**
USE***
conv***
fc*
conv*
conv
USE**

Fig C. Layer-wise and dimension-wise relations of Deep Neural Network representations to human behavioral similarity judgements. (A) We tested whether similarity judgements in the respective task-relevant perceptual and conceptual spaces corresponded to representational distances in specific DNN layers. To this end, we computed the similarity of each image pair in each network layer of a convolutional DNN and in the universal sentence encoder (USE) and used these similarities as predictors in a linear mixed model on rated distances of Studies I and II. (B) To exclude effects of the chosen similarity metric we repeated the layerwise analysis using Pearson correlation and obtained the same results as using Spearman rho to compare the subject matrices to the DNN matrices. Indeed, we replicated our results that cDNN layers conv3 ($t_{101} = 2.71$, $p = 0.017$, $d = 0.21$, perceptual: 0.074±0.10, conceptual: 0.04±0.09), conv4 ($t_{101} = 2.30$, $p = 0.025$, $d = 0.23$, perceptual: 0.07±0.10, conceptual: 0.04±0.09) and conv5 ($t_{101} = 2.71$, $p = 0.017$, $d = 0.27$, perceptual: 0.08±0.09, conceptual: 0.05±0.09) predicted perceptual ratings better than conceptual ratings, while the reverse was true for fully-connected layer 8 ($t_{101} = -2.51$, $p = 0.020$, $d = 0.25$, perceptual: 0.10±0.10, conceptual: 0.14±0.11) and USE distances ($t_{101} = -2.80$, $p = 0.017$, $d = 0.28$, perceptual:0.04±0.10, conceptual: 0.08±0.09). (C) Comparing the fit of USE similarities to participant ratings for each of the conceptual dimensions separately, we find that ratings for real-world size are reflected best in USE similarities (size vs. approach: $t_{101} = 3.65$, $p<0.001$; size vs. climate: $t_{101} = 4.88$, $p<0.001$; climate vs. approach: $t_{101} = -1.13$, $p = 0.783$; corrected for multiple comparisons). Testing the overall USE – participant fit (t-test against zero), we find two of three dimensions to be significantly related to participant judgements (size: $t_{101} = 9.66$, $p<0.001$; approach: $t_{101} = 3.13$, $p = 0.014$; climate: $t_{101} = 1.55$, $p = 0.75$; corrected for multiple comparisons). (D) Dimension-wise ratings and DNN metrics. Top: Histograms of participant response ratings demonstrating the use of the full rating range during encoding. Bottom: Layerwise Spearman correlation to rated distances from the respective dimension. Left: Linear mixed models using DNN averages and the semantic model (see Method section for details) to predict participant ratings separately for each dimension (**size**: conv: $z = 4.03$, $\chi^2_{(1)} = 16.22$, $p < 0.001$; fc: $z = -22.31$, $\chi^2_{(1)} = 477.21$, $p < 0.001$; USE: $z = -5.70$, $\chi^2_{(1)} = 32.48$, $p < 0.001$; **climate**: conv: $z = -2.83$, $\chi^2_{(1)} = 8.03$, $p = 0.004$; fc: $z = -2.16$, $\chi^2_{(1)} = 4.70$, $p = 0.030$; USE: $z = -4.21$, $\chi^2_{(1)} = 17.70$, $p < 0.001$; **approach**: conv: $z = 1.90$, $\chi^2_{(1)} = 3.62$, $p = 0.056$; fc: $z = -4.81$, $\chi^2_{(1)} = 23.14$, $p < 0.001$; USE: $z = -3.12$, $\chi^2_{(1)} = 9.77$, $p = 0.002$; **color:** conv: $z = -7.00$, $\chi^2_{(1)} = 48.90$, $p < 0.001$; fc: $z = -5.18$, $\chi^2_{(1)} = 26.85$, $p < 0.001$; USE: $z = -1.91$, $\chi^2_{(1)} = 3.68$, $p = 0.055$; **camouflage**: conv: $z = -1.49$, $\chi^2_{(1)} = 2.24$, $p = 0.134$; fc: $z = -9.07$, $\chi^2_{(1)} = 81.77$, $p < 0.001$; USE: $z = -1.07$, $\chi^2_{(1)} = 1.16$, $p = 0.281$; **orientation**: conv: $z = 0.02$, $\chi^2_{(1)} = 0.00$, $p = 0.980$; fc: $z = -5.85$, $\chi^2_{(1)} = 34.22$, $p < 0.001$; USE: $z = -0.71$, $\chi^2_{(1)} = 0.52$, $p = 0.473$; see Supplementary Table 2 for detailed linear mixed model statistics). Data are visualized after removing participant-wise estimated random effects. 95% confidence intervals, error bars indicate SEM. *, $p<0.05$, **, $p<0.01$, ***, $p<0.001$

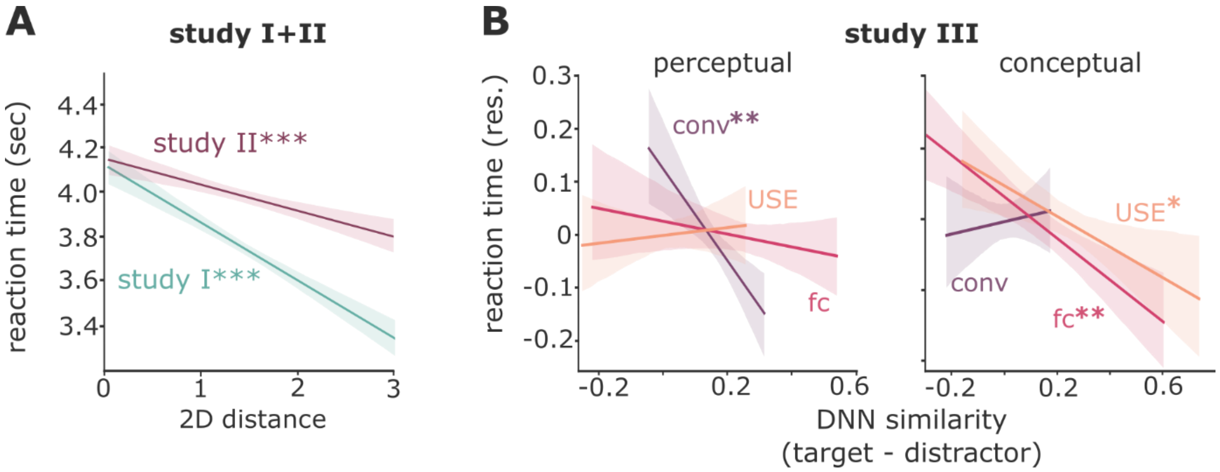

Fig D: Reaction times are influenced relate to DNN distances: (A) We first tested whether representational distances affected the ease of similarity judgments – i.e., whether responses to images were given faster when their distances on relevant dimensions were larger. We used a linear mixed model to test the effect of task-relevant distance on reaction times including a random participant intercept. Significance testing was done using a likelihood ratio test between a full model, including the fixed effect of interest, and a reduced model without the fixed effect of interest (participant intercept only). Unsurprisingly, we found that larger 2D distances of task-relevant dimensions predicted faster response times (Study I: $z = -11.28$, $\chi^2_{(1)} = 126.69$, $p < 0.001$; Study II: $z = -6.67$, $\chi^2_{(1)} = 44.49$, $p < 0.001$). (B) Similar to Studies I and II, we found higher distances to predict faster response times. Response times during perceptual choices were predicted in a linear mixed model with a random participant intercept using distances in low-level perceptual space ($z = -3.90$, $\chi^2_{(1)} = 15.20$, $p = 0.001$; likelihood ratio test) but not higher-order perceptual space ($z = -1.29$, $\chi^2_{(1)} = 1.67$, $p = 0.196$) or semantic space ($z = 0.46$, $\chi^2_{(1)} = 0.22$, $p = 0.639$; likelihood ratio test). Contrastingly, response times during conceptual choices were predicted by distances in both higher-order perceptual ($z = -4.91$, $\chi^2_{(1)} = 24.00$, $p < 0.001$; likelihood ratio test) and semantic spaces ($z = -2.68$, $\chi^2_{(1)} = 7.21$, $p = 0.007$; likelihood ratio test), but not low-level perceptual space ($z = 0.49$, $\chi^2_{(1)} = 0.24$, $p = 0.621$; likelihood ratio test).

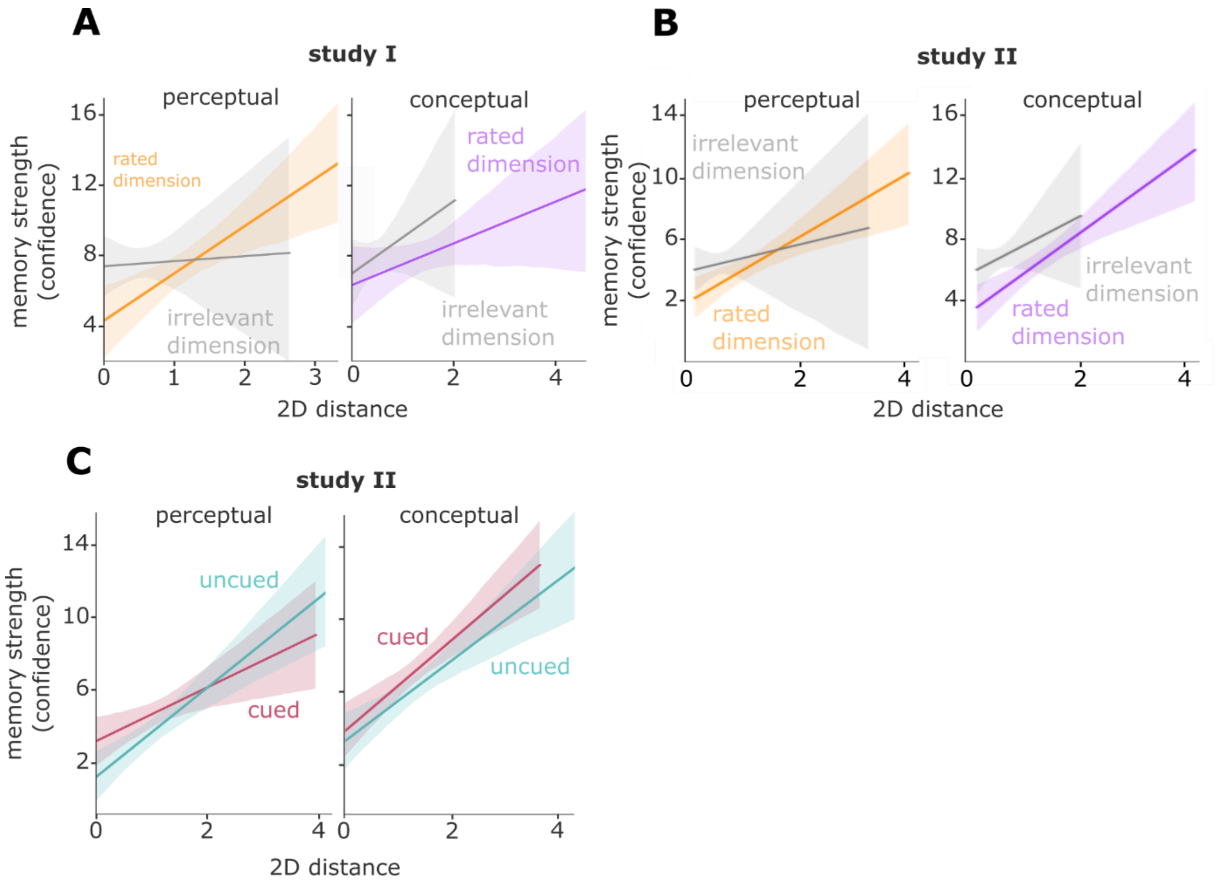

Fig E: Representational distance affects subsequent memory regardless of perceptual/conceptual encoding task and cueing. (A) Study I: Encoding task did not affect the impact of representational distance on subsequent memory (interaction space x rated distance: z = 1.56, $\chi^2_{(2)}$ = 2.89, p = 0.235). (B) Same for Study II (interaction space x rated distance: z = -0.63, $\chi^2_{(2)}$ = 0.40, p = 0.820). (C) Study II: Cueing did not affect the impact of representational distance neither for perceptual (interaction cueing x rated distance [perceptual trials only]: z = 1.14, $\chi^2_{(2)}$ = 1.30, p = 0.52) nor for conceptual encoding tasks (interaction cueing x rated distance [conceptual trials only]: z = -0.11, $\chi^2_{(2)}$ = 0.01, p = 0.99). Data are visualized after removing participant-wise estimated random effects. 95% confidence intervals.

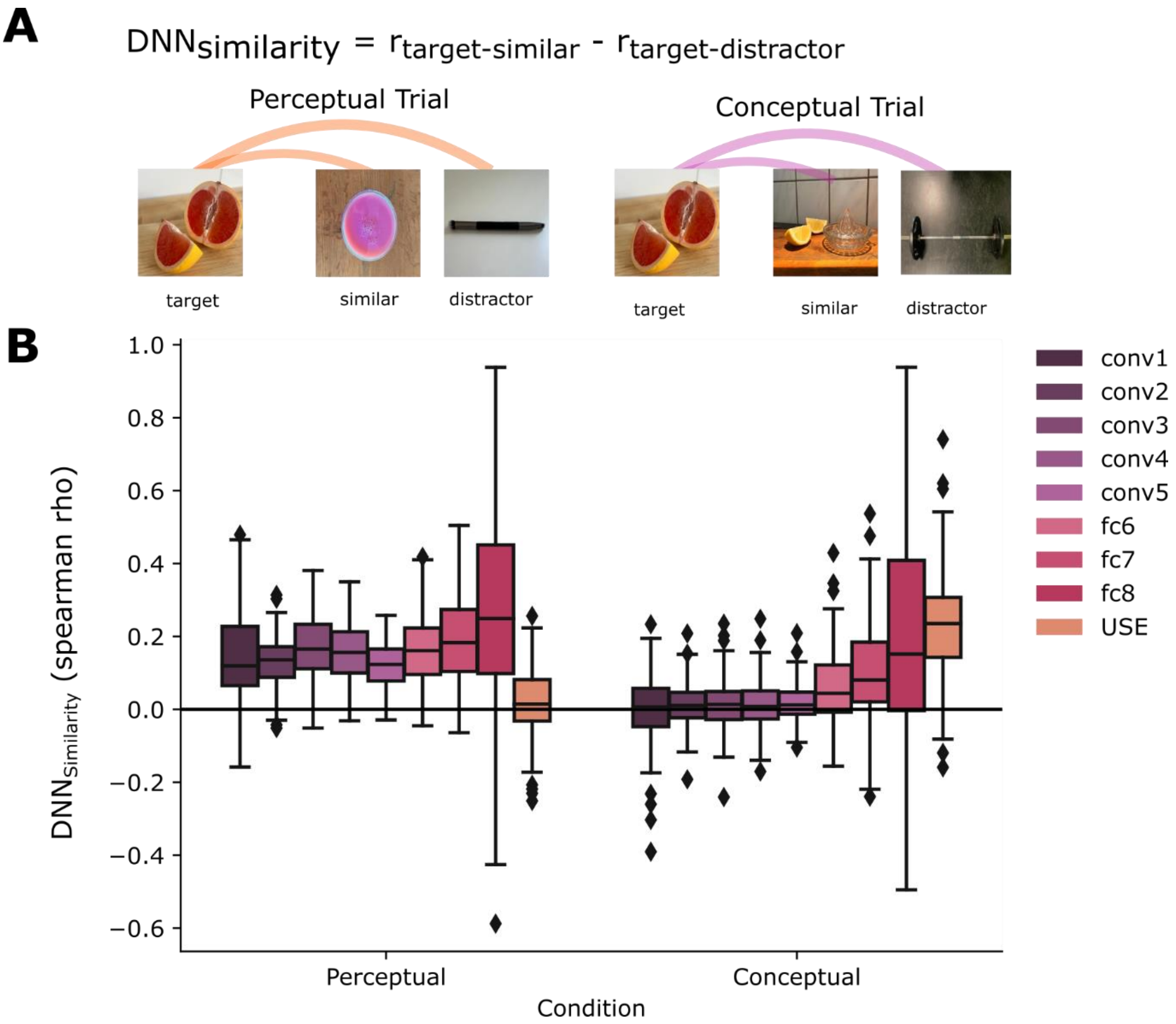

Fig F: Layer-wise correspondence of representational distances in DNNs for triplets in Study III. (A) Here, we computed similarities scores based on DNN model similarity matrices only since for Study III we only acquired binary ratings indicating which image was rated as more similar to the target. Exemplary target image with perceptual and conceptual choice options (similar choice, distractor choice). Lines indicate pairwise image similarities used to compute a triplet similarity score ($d_{similarity}$). (B) Distribution of $d_{similarity}$ scores across cDNN layers for conceptual and perceptual trials. Values larger than zero indicate higher DNN-based representational similarities of targets with the similar image as compared to the distractor image. For each layer and condition, boxplots depict the distribution of $d_{similarity}$ scores across triplets. For perceptual triplets, $d_{similarity}$ scores are significantly larger than zero for all cDNN layers, but not for the USE. For conceptual triplets, $d_{similarity}$ scores are significantly larger than zero from conv2 until and including the USE model. Center line indicates median, box limits indicate upper and lower quartiles, whiskers indicate 1.5 interquartile range, points represent outliers.

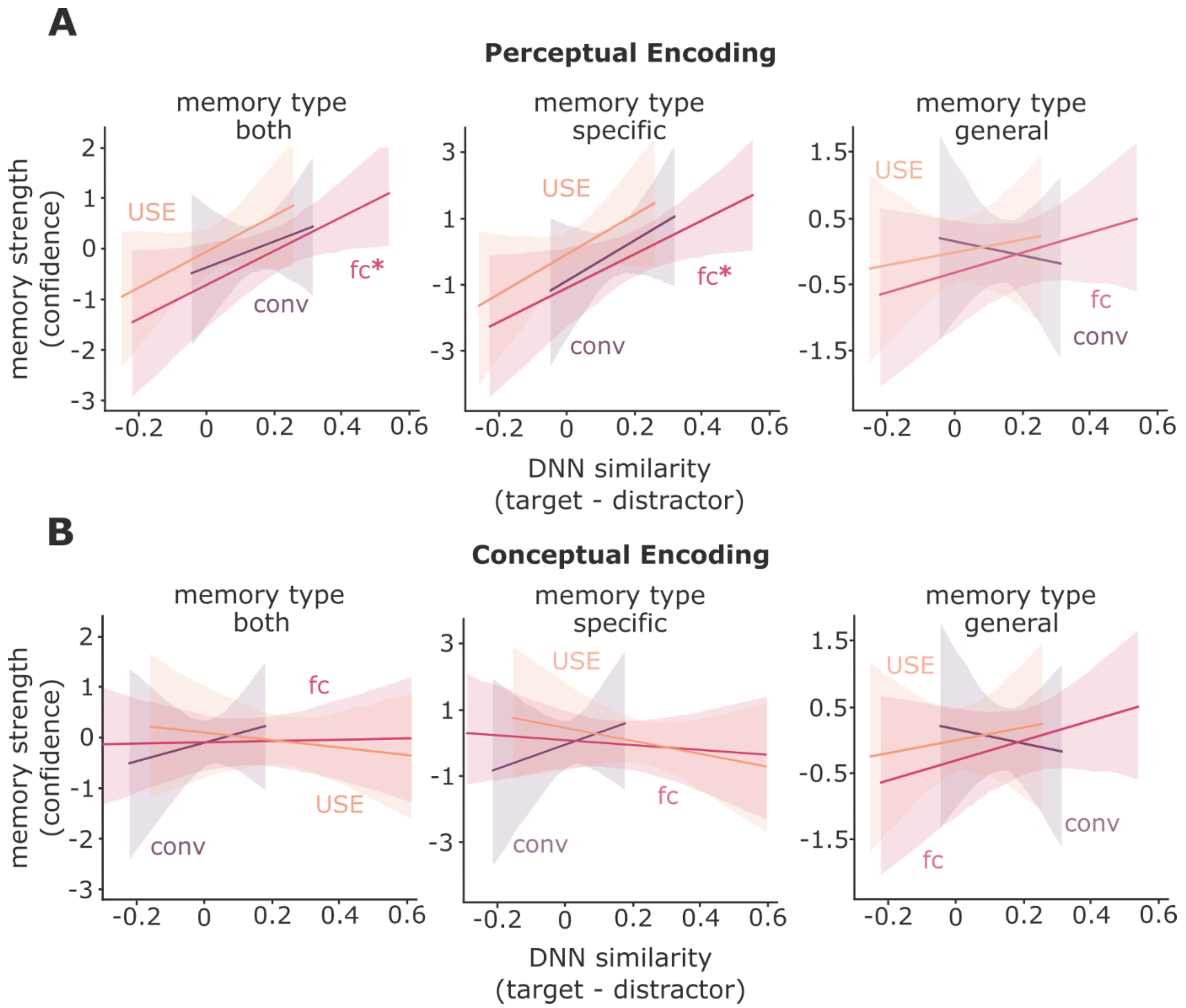

Fig G: Effects of DNN-based representational similarities on memory strength during the general and the specific memory task. (A) Prediction of memory strength by DNN similarities during perceptual choices. Left: Collapsed across memory types, representational distances in fully-connected layers but not convolutional layers (conv) and not semantic distances (USE-based) predict memory strength (see Results section). Middle: Memory strength in the specific retrieval task is predicted by fully-connected (: z = 2.258, $\chi^2_{(1)} = 5.09$, p = 0.024) but not convolutional or semantic (conv: z = 1.138, $\chi^2_{(1)} = 1.29$, p = 0.255; USE: z = 1.567, $\chi^2_{(1)} = 2.45$, p = 0.117; likelihood ratio test) DNN similarities during perceptual encoding. Right: Memory in the general memory test is independent of DNN similarities during perceptual encoding (conv: z = -0.293, $\chi^2_{(1)} = 0.09$, p = 0.769; fc: z = 0.977, $\chi^2_{(1)} = 0.95$, p = 0.328; USE: z = 0.373, $\chi^2_{(1)} = 0.14$, p = 0.709). (B) Prediction of memory strength by DNN similarities during conceptual encoding. Left: Collapsed across memory types, DNN-predicted similarities do not predict subsequent memory (see Results section). Middle: Memory strength during specific retrieval cannot be predicted by DNN similarities (conv: z = 0.667, $\chi^2_{(1)} = 0.45$, p = 0.504; fc: z = -0.404, $\chi^2_{(1)} = 0.16$, p = 0.686; USE: z = -0.807, $\chi^2_{(1)} = 0.65$, p = 0.419; likelihood ratio test). Right: Memory strength during general retrieval cannot be predicted by DNN similarities. For studies I and II, where task-demands did not differ between general and specific retrieval, we found no effect of DNN similarities on memory strengths (Study I+II: conv: z = 0.49, $\chi^2_{(1)} = 1.34$, p = 0.247; fc: z = 0.94, $\chi^2_{(1)} = 1.00$, p = 0.317; USE: z = 0.87, $\chi^2_{(1)} = 1.90$, p = 0.167; likelihood ratio test). Data are visualized after removing participant-wise estimated random effects. 95% confidence intervals. *, p<0.05

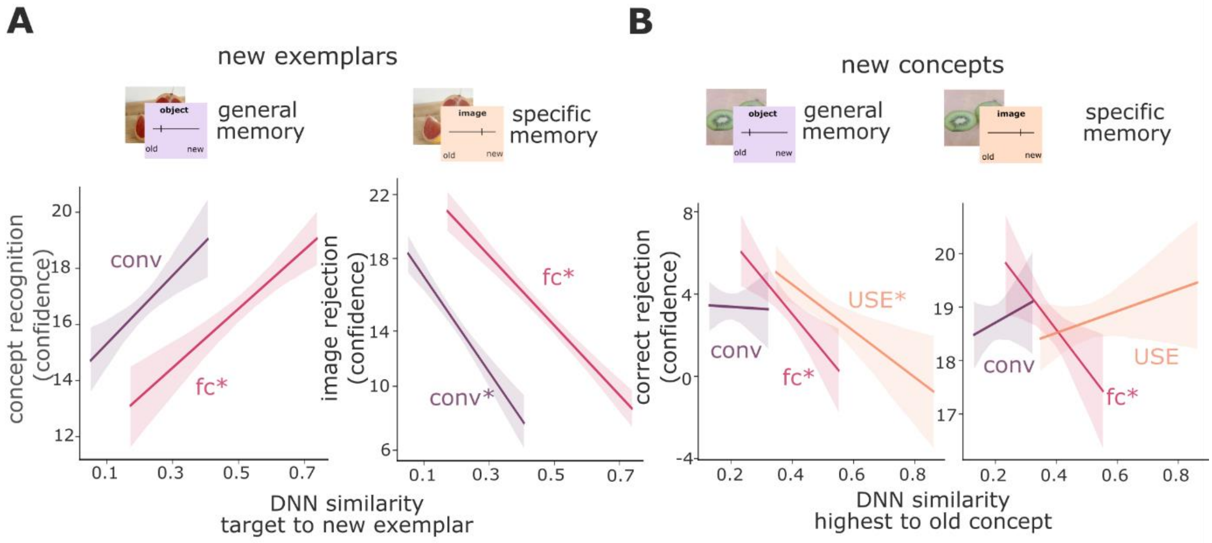

Fig H: Different formats contribute to memory strength and correct rejection performance depending on task instructions at retrieval. (A) We computed the similarity of each new exemplar image to the old image of the same category and extracted the similarity value as the DNN similarity (for each DNN layer). Using a linear mixed model including both the main effects of DNN layers and task, we found significant effects of retrieval instruction with distances in low-level perceptual space (z = -2.22, $\chi^2_{(1)}$ = 13.30, p = 0.0002; likelihood ratio test) and higher-order perceptual space (z = -7.89, $\chi^2_{(1)}$ = 70.34, p<0.0001; likelihood ratio test) indicating that representational distances between new and previously presented items exerted different effects depending on retrieval tasks. Correct rejection performance for new exemplars in the specific retrieval task depended on similarities of both low-level perceptual (z =-2.51, $\chi^2_{(1)}$ = 6.34, p = 0.011; likelihood ratio test) and higher-order perceptual (z = -7.16, $\chi^2_{(1)}$ = 50.97, p<0.0001; likelihood ratio test) formats, while memory strength (confidence) for old concepts during general retrieval depended on similarity of higher-order (z = 4.40, $\chi^2_{(1)}$ = 19.37, p<0.0001; likelihood ratio test) but not low-level perceptual formats (z = 0.51, $\chi^2_{(1)}$ =0.26, p = 0.6090; likelihood ratio test). (Note that semantic formats could not be considered in this analysis as new exemplars always belonged to the same concept.) (B) For new concepts we calculated the semantic similarity to all encoding concepts and extracted the highest value (highest similarity of new concept – all old images/concepts): Using a linear mixed model including DNN similarities as predictors, we found that higher-order perceptual (z =-2.78, $\chi^2_{(1)}$ = 7.73, p = 0.005; likelihood ratio test) but not low-level perceptual (z =1.39, $\chi^2_{(1)}$ =1.95, p = 0.162; likelihood ratio test) or USE (z =0.50, $\chi^2_{(1)}$ = 0.25, p = 0.615; likelihood ratio test) similarities predicted performance during specific retrieval. During general retrieval, both higher-order perceptual (z =-4.31, $\chi^2_{(1)}$ = 18.57, p < 0.0001; likelihood ratio test) and semantic similarities (z =-3.96, $\chi^2_{(1)}$ = 15.65, p < 0.0001; likelihood ratio test) but not low-level perceptual similarities (z =1.14, $\chi^2_{(1)}$ = 1.32, p = 0.250; likelihood ratio test) predicted rejection of new concepts. Data are visualized after removing participant-wise estimated random effects. 95% confidence intervals. *, p<0.05

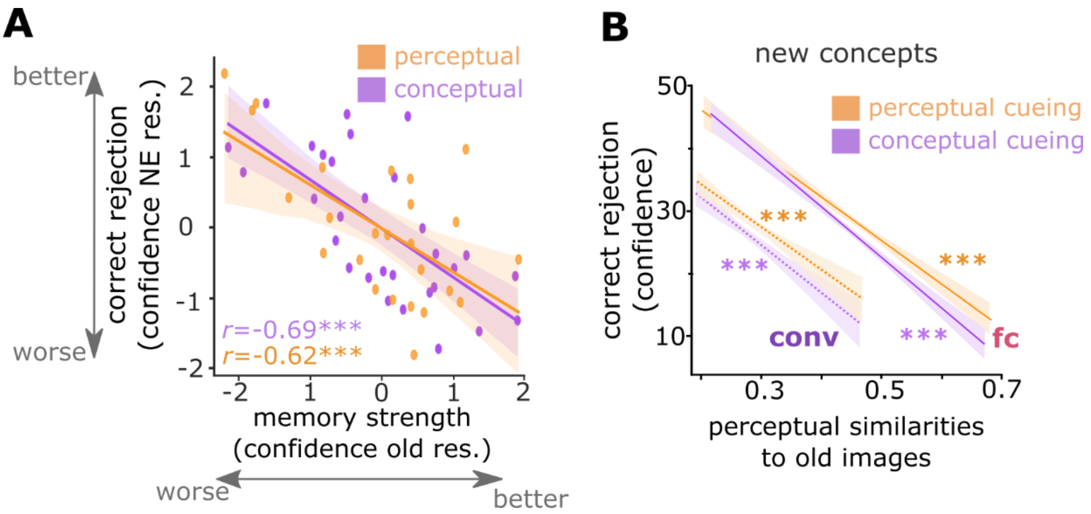

Fig I: Conceptual cueing specifically influences semantic memory. (A) We tested whether participants who showed higher recognition memory performance also showed a decrease in rejection performance of new exemplars because they generally used a more liberal criterion during memory decisions. To this end, we calculated Pearson's correlations on participants' mixed model residuals. We found that participants in both conceptual (r = -0.69, p<0.001) and perceptual (r = -0.62, p<0.001) cueing conditions show this effect, i.e., those who performed better in identifying old images made more errors for new exemplars. (B) Since conceptual cueing impaired the rejection of new concepts that were semantically highly similar to old concepts, we tested whether cueing affected the rejection of perceptually similar new concepts as well. To this end – analogously to our analysis on semantic similarities (Fig. 5d) – we computed two linear mixed models (one for perceptual cueing and one for conceptual cueing) using perceptual similarities from the cDNN as predictors (highest similarity new concept – old images). Perceptual features predicted correct rejection confidence in both perceptual (conv: z = -6.64, $\chi^2_{(1)}$ = 43.84, p < 0.001; fc: z = -13.71, $\chi^2_{(1)}$ = 183.88, p < 0.001) and conceptual cueing conditions (conv: z = -7.21, $\chi^2_{(1)}$ = 51.69, p < 0.001; fc: z = -15.46, $\chi^2_{(1)}$ = 233.53, p < 0.001). Thus, low-level perceptual (conv) and higher-order perceptual (fc) similarities impaired rejection performance following both perceptual and conceptual cueing. Data are visualized after removing participant-wise estimated random effects. 95% confidence intervals. ***, p<0.001

Table A: Linear mixed models and their beta weights from main results. Corresponding Beta weights to the z values reported in the main results. All models include a random participant intercept (1|participant).

Linear mixed model	β	*p*	Linear mixed model	β	*p*
**Rated distances predicted by DNN layer (SI+II)**			**Confidence for old images by encoding space using DNNs (SIII)**		
Rated distance ~ conv + fc + USE (perceptual encoding)			*Memory confidence ~ conv + fc + USE (perceptual encoding)*		
Conv	-0.47	<0.001	Conv	2.91	0.460
Fc	-0.44	<0.001	Fc	3.91	0.022
USE	-0.09	0.060	USE	3.71	0.151
Rated distance ~ conv + fc + USE (conceptual encoding)			*Memory confidence ~ conv + fc + USE (conceptual encoding)*		
Conv	0.20	0.078	Conv	2.32	0.597
Fc	-0.67	<0.001	Fc	0.16	0.913
USE	-0.40	<0.001	USE	-0.76	0.632
			**Confidence for old images by encoding space (SIII)**		
			Memory confidence ~ encoding type + memory type + encoding space*memory type		
**Confidence for old images by encoding space (SI+SII)**			encoding space*memory type	1.64	0.007
Memory confidence (old images) ~ encoding space (**I**)	-0.22	0.716	*Memory confidence ~ encoding space (specific memory)*	0.66	0.198
Memory confidence (uncued old images) ~ encoding space (**II**)	0.49	0.875	*Memory confidence ~ encoding space (general memory)*	-0.98	0.002
**Confidence for old images by 2D distance (SI+II)**			**Confidence for new exemplars by encoding space (SIII)**		
Memory confidence ~ relevant distance (**I**)	1.76	<0.001	_Confidence ~ encoding type + memory type + encoding space *memory type_		
Memory confidence ~ relevant distance (**II**)	2.10	<0.001	encoding space*memory type	1.52	0.011
Memory confidence ~ irrelevant distance + relevant distance (**I**)	0.72	0.431	*Rejection confidence ~ encoding space (specific memory)*	-0.17	0.680
Memory confidence ~ irrelevant distance + relevant distance (**II**)	0.95	0.332	*Memory confidence ~ encoding space (general memory)*	-1.73	<0.001

Table A continued

**Confidence for old images by 2D distance depending on temporal order**		
Memory confidence (first image) ~ relevant distance (**I**)	0.98	0.100
Memory confidence (second image) ~ relevant distance (**I**)	2.51	<0.001
Memory confidence (first image) ~ relevant distance (**II**)	2.37	<0.001
Memory confidence (second image) ~ relevant distance (**II**)	2.27	<0.001
**TMR effects on confidence for old images (SII)**		
Memory confidence (old images) ~ encoding space + cueing		
Encoding space	1.66	0.552
Cueing	1.04	0.033
*Memory confidence (old images) ~ encoding space + cueing + encoding space*cueing*		
Encoding space*Cueing	4.31	<0.001
Memory confidence (old images) ~ cueing (cued conceptual encoding)	3.00	<0.001
Memory confidence (old images) ~ cueing (cued perceptual encoding)	1.30	0.060
**Reaction Times by rated distance (SI-II)**		
Reaction times ~ rated distance 2D (**I**)	-277.82	<0.001

**Rejection confidence for new exemplars by 2D distance (SI+II)**		
Rejection confidence ~ relevant distance (**I**)	-0.83	0.129
Rejection confidence ~ relevant distance (**II**)	-0.36	0.434
**TMR effects on rejection confidence for new exemplars and new concepts (SII)**		
Rejection confidence (new exemplars) ~ encoding space + cueing		
Encoding space	1.20	0.684
Cueing	0.024	0.969
*Rejection confidence (new exemplars) ~ encoding space + cueing + encoding space*cueing*		
Encoding space*cueing	4.62	<0.001
Rejection confidence (new exemplars) ~ cueing (cued conceptual encoding)	2.12	0.017
Rejection confidence (new exemplars) ~ cueing (cued perceptual encoding)	-2.49	0.006
Rejection confidence (new concepts) ~ cueing (cued conceptual encoding)	-12.34	0.008
Rejection confidence (new concepts) ~ cueing (cued perceptual encoding)	-7.11	0.109
Rejection confidence (new exemplars, uncued) ~ encoding space	1.10	0.726
Reaction times ~ rated distance 2D (**II**)	-148.62	<0.001

Table B: Linear mixed models and their beta weights from Supplementary Analyses. Corresponding Beta weights to the z values reported in the Supplementary Analyses. All models include a random participant intercept (1|participant).

Linear mixed model	β	*p*	Linear mixed model	β	*p*
**Supplementary Fig. A: Reaction times by distance in mixed vs congruent blocks (SI)**					
Reaction times (perceptual trials) ~ rated distance + congruency (mixed/congruent)			*Reaction times (conceptual trials) ~ rated distance + congruency* (mixed/congruent)		
Congruency	67.18	0.099	Congruency	59.35	0.167
**Supplementary Fig. A: Confidence for old images by space (SI)**			**Supplementary Fig. A: Rejection confidence for new exemplars by space (SI)**		
Memory confidence ~ space			*Rejection confidence ~ space*		
Mixed (vs conceptual)	-0.17	0.777	Mixed (vs conceptual)	0.08	0.915
Perceptual (vs conceptual)	0.22	0.716	Perceptual (vs conceptual)	1.41	0.082
Perceptual (vs mixed)	0.40	0.518	Perceptual (vs mixed)	1.32	0.102
**Supplementary Fig. E: Confidence for old images by 2D distance and space (SI)**			**Supplementary Fig. E: Confidence for old images by 2D distance and cueing (SI+II)**		
*Memory confidence ~ 2D distance + space + 2D distance*space*			*Memory confidence ~ 2D distance + 2D distance*cueing (perceptual cueing)*		
2D*perceptual (vs conceptual)	1.64	0.118	2D*cueing	0.89	0.254
2D*mixed (vs conceptual)	0.16	0.874	*Memory confidence ~ 2D distance + 2D distance*cueing (conceptual cueing)*		
2D*mixed (vs perceptual)	1.47	0.167	2D*cueing	-0.08	0.911
**Supplementary Fig. D: Reaction Times by DNN similarity (SI-III)**					
Reaction times ~ rated distance 2D (**I**)	-277.82	<0.001	*Reaction times ~ rated distance 2D* (**II**)	-148.62	<0.001
*Reaction times ~ conv + fc + USE (**III**: perceptual encoding)*			*Reaction times ~ conv + fc + USE (III: conceptual encoding)*		
Conv	-0.47	<0.001	Conv	0.20	0.078
Fc	-0.44	<0.001	Fc	-0.67	<0.001
USE	-0.09	0.060	USE	-0.40	<0.001

Table B continued

Supplementary Fig G: Confidence by space and retrieval task (SIII)

Memory confidence (old images) ~ conv + fc + USE (perceptual encoding|specific memory)			*Memory confidence (old images) ~ conv + fc + USE* (conceptual encoding|specific memory)		
Conv	7.11	0.255	Conv	4.69	0.504
Fc	6.11	0.024	Fc	-0.95	0.686
USE	6.41	0.117	USE	-2.04	0.420
Memory confidence (old images) ~ conv + fc + USE (perceptual encoding|general memory)			*Memory confidence (old images) ~ conv + fc + USE* (conceptual encoding|general memory)		
Conv	-1.21	0.770	Conv	-0.03	0.993
Fc	1.74	0.329	Fc	1.28	0.347
USE	1.00	0.709	USE	0.52	0.718

Supplementary Fig. H: Confidence for new exemplars and new concepts by encoding space and retrieval task (SIII)

Memory confidence (new exemplars) ~ conv + fc (general memory)			*Rejection confidence (new exemplars) ~ conv + fc* (specific memory)		
Conv	1.63	0.609	Conv	-9.08	0.012
Fc	7.90	<0.001	Fc	-14.51	<0.001
Rejection confidence (new concepts) ~ conv + fc + USE (general memory)			*Rejection confidence (new concepts) ~ conv + fc + USE* (specific memory)		
Conv	6.67	0.250	Conv	4.61	0.163
Fc	-17.92	<0.001	Fc	-6.57	0.005
USE	-10.96	<0.001	USE	0.79	0.616

Supplementary Fig. I: Rejection confidence of new concepts depending on highest perceptual similarity (new concept – all old images) and cueing (SII)

Rejection confidence (perceptual cueing) ~ conv + fc			*Rejection confidence (conceptual cueing) ~ conv + fc*		
Conv	-186.33	<0.001	Conv	-239.82	<0.001
Fc	-81.31	<0.001	Fc	-89.22	<0.001

Table B continued

Supplementary Fig. C: DNN prediction of rating responses for each single dimension

Ratings (size) ~ conv + fc + USE			*Ratings (color) ~ conv + fc + USE*		
Conv	4.03	<0.001	Conv	-7.00	<0.001
Fc	-22.31	<0.001	Fc	-5.18	<0.001
USE	-5.70	<0.001	USE	-1.91	0.055
Ratings (approach) ~ conv + fc + USE			*Ratings (camouflage) ~ conv + fc + USE*		
Conv	1.90	0.057	Conv	-1.49	0.134
Fc	-4.18	<0.001	Fc	-9.07	<0.001
USE	-3.12	0.002	USE	-1.07	0.282
Ratings (climate) ~ conv + fc + USE			*Ratings (orientation) ~ conv + fc + USE*		
Conv	-2.83	0.005	Conv	0.025	0.980
Fc	-2.16	0.030	Fc	-5.89	<0.001
USE	-4.21	<0.001	USE	-0.71	0.473
