# Supplementary material for: Task-relevant representational spaces in human memory traces
Source: PLoS Comput Biol. 2025 Sep 12;21(9):e1013476. doi: 10.1371/journal.pcbi.1013476 (PMC12448971; doi:10.1371/journal.pcbi.1013476)
Supplement: Attachment — Submitted filename: Heinen_et_al_PLOScompBio_responseletter.docx [file pcbi.1013476.s002.docx]

Dear Prof. Bays,

Thank you for giving us the opportunity to revise our manuscript, entitled " Task-relevant representational spaces in human memory traces" (Manuscript Number: PCOMPBIOL-D-25-00496). We also wish to thank the two reviewers for their thoughtful comments and suggestions. We have carefully examined all the issues that were raised and have revised the manuscript accordingly.

Our responses to all the issues raised by the reviewers and the modifications that have been made in the manuscript are described below. These modifications are highlighted in green in the revised (colored file version) manuscript. We also changed the issues raised regarding the journal requirements in the manuscript and the submission system.

# **Response to reviewers:**

# **Reviewer #1**

**Heinen et al. use a behavioral and modelling approach to show that memories are encoded into representational spaces whose formats are dependent on task demands. Notably, representations derived from DNNs correlate with behavioral judgments where distances within task-relevant representational spaces influence memory. This is an impressive and well-executed study that offers valuable insights into the nature of human memory representations using an combination of behavioral experiments and deep neural network modeling. The analyses are thorough, the experimental design is clever, and the manuscript is clearly written, making a strong contribution to our understanding of computational cognitive neuroscience. I mainly have a few clarification questions and suggestions below.**

We thank the Reviewer for the overall positive evaluation of our work and are pleased that the usefulness of DNN representations in the study of human memory was well received. We carefully considered each individual point raised by the reviewer and believe that this has substantially improved the quality of the article.

**1. The Nature of Representational Space - metric?: Do these spaces strictly adhere to metric properties? Do the authors think there is a real metric space we're all using, or is it more individualized, and doesn't matter exactly what number it is? The typical use of rank correlations in RSA suggests this it might not matter exactly what the metric is.**

We agree that this is a highly relevant point that we carefully addressed in the introduction and discussion section of the revised manuscript. Further, we included additional analyses to 1) quantify the similarity between participant-specific and global spaces and 2) demonstrate that comparable results are obtained when using Pearson’s instead of Spearman’s rank correlation.

In the introduction section of the revised version of the manuscript we now explicitly address characteristics of these spaces, in direct reference to metric relationships between the elements within these spaces:

“*Since the relation between concepts and items in an abstract space is measured by the distance between the concepts, previous studies suggest the underlying maps to adhere to metric properties (Bellmund et al. 2018). However, it remains to be elucidated whether task properties or interindividual differences in participant behavior create or influence these metrics. Thus, if participants rely on a metric cognitive space, similarities in abstract spaces should not only be present for metric task instructions but also in binary ratings. Information in cognitive spaces should also allow for generalization and inference to new concepts in the form of shortcuts. If we actually access abstract spaces depending on different representational formats, similarities in these spaces should be behaviorally relevant. In addition, idiosyncratic vs. shared (i.e., individual vs. collective) similarity structure in abstract cognitive maps remains to be explored.*” (p. 3, line 91-101)

Regarding idiosyncrasies in representational spaces, we added an additional analysis to the results section of the revised manuscript. Specifically, we quantified the similarity of each individual space to the reconstructed global map (i.e. a reconstructed space based on all ratings across the sample), demonstrating that perceptual ratings seem to be more individually biased than conceptual ratings (Fig. 3D, former Fig. 2).

**Fig. 3D:** Subject to group level space correlations demonstrate how well the individual maps reflect the global space. Perceptual spaces show more individual differences, compared to conceptual spaces which appear more general across participants.

In the discussion, we further address this point by offering alternative explanations (graph-based spaces) and reflect on their relevance for our studies and results as well as future studies on global and individual spaces:

“*While our study suggests that information represented in abstract spaces may be metric, it is possible that other measures of representational geometry are at play. For instance, graph-like representational structures (Garvert et al. 2017) might be used in our task, although ultimately these may also employ a metric (i.e., two steps on the graph are equivalent to a certain distance). The use of a visual scale might have enforced the use of a metric space in our task; however, we found similar results for Study III where participants indicated similarity via binary responses. It would also be interesting to see if the spaces in our study enable shortcuts by predicting relations for leave-out concepts. Even more interesting is the overall fit of the individual spaces to the global group space, indicating an overlap between interindividual similarity judgements and a collective representational structure in the employed abstract maps. Specifically we find that perceptual spaces were more individualized than conceptual spaces, which is supported by a recent finding suggesting that perceptual ratings are highly subjective and individually different (Kawakita et al. 2025). The more conceptual or abstract these spaces become, the more similar they might be across people. This raises exciting new questions about the nature of interindividual differences in representational spaces – will conceptual spaces become more similar to a global group space during development, as individuals learn and interact with these concepts? Which factors (i.e., prior knowledge, attention, reward, etc. influence how similar your own space is to the global map?“* (p. 20, line 617-634)

Finally, we replicate the findings from the analysis of model-human fit using Pearson’s instead of Spearman’s rank correlation, suggesting that representational spaces are indeed organized by metric distances, allowing shortcuts between arbitrary concept pairs (Supplementary Fig. C).

**Supp. Fig. C:** (B) To exclude effects of the chosen similarity metric we repeated the layerwise analysis using Pearson correlation and obtained the same results as using Spearman rho to compare the subject matrices to the DNN matrices.

**2. Surprisingly good human-model link given the task: Conceptual space in humans in this study is distance based on size and climate (2D). In the DNN space it is distance in general (high dimensional). How is it that only two conceptual features is surprisingly similar to the DNN's general distance presumably with many semantic features? I understand it probably captures part of the variance, but do you think you're capturing most of it, or just a part of it?**

We thank the reviewer for this comment and added more information and details on the DNN similarities. First of all, we want to point out that we have three conceptual distance dimensions in our study: size (how big is the animal in real life), climate (hot or cold climate) and approach (would you approach or avoid this animal?). While we initially only reported the similarity of the DNNs to the overall perceptual and conceptual spaces, we now extend these findings by showing human-DNN model fit for each conceptual and perceptual rating dimension separately (Supplementary Fig. C).

**Supp. Fig. C.**:(D) Dimension-wise ratings and DNN metrics. Top: Histograms of participant response ratings demonstrating the use of the full rating range during encoding. Bottom: Layerwise Spearman correlation to rated distances from the respective dimension. Left: Linear mixed models using DNN averages and the semantic model (see Method section for details) to predict participant ratings separately for each dimension (see Supplementary Table B for linear mixed model statistics).

And we refer to the dimension-wise DNN relations in the results:

*In addition, we tested how the DNN similarities reflected human ratings for each rating dimension separately (see Supplementary Fig. C in S1 Text for details and results).”* (p. 6, line 203-205)

Indeed, not all dimensions relate to the DNNs to the same degree. Especially (real world) size seems to relate to late cDNN layers and the semantic model. To this end, we computed t-tests on the participant average correlations to the USE model between each of the three conceptual dimensions. We find that size can be explained by USE similarities better than approach and climate (Supplementary Fig. C). Using the average participant – USE model correlations for each conceptual dimension we show that we cover only a small part of the variance (size: rho_mean_ = 0.135, R^2^ = 0.018; approach: rho_mean_ = 0.054, R^2^ = 0.002; climate: rho_mean_ = 0.026, R^2^ = 0.0006; sum over dimensions: R^2^ = 0.0206) which however is significant for two of the three dimensions (size: t_101_ = 9.66, p<0.001; approach: t_101_ = 3.13, p = 0.014; climate: t_101_ = 1.55, p = 0.75; corrected for multiple comparisons). Also, in the dimension-wise linear mixed models we find the same effect of conceptual dimensions being predicted by fc and USE and the perceptual dimensions being predicted by conv and fc. We added this to the Supplementary Fig. C. However, we think that we only capture a part of the variance: A) We specifically ask for three sub-dimensions (i.e., climate, approach, size), but obtaining insights into the overall variance that can be covered by semantic models would require a much higher number of different dimensions to be tested. B) Language models are trained on the human semantic space and understanding of the world by learning stochastic relationships between words / concepts.). Importantly, one characteristic of this space is that a lot of features are not necessarily orthogonal (i.e., an animal that is smaller will with a high probability also be easy and safe to approach). In addition, the names of animals with similar features tend to appear together more often in the text corpora used to train the networks. Taken together, we think we can only capture part of the variance that the abstract concept space offers (and consists of).

**Supp. Fig. C.**:(C) Comparing the fit of USE similarities to participant ratings for each of the conceptual dimensions separately, we find that ratings for real-world size are reflected best (size vs. approach: t101 = 3.65, p<0.001; size vs. climate: t101 = 4.88, p<0.001; climate vs. approach: t101 = -1.13, p = 0.783; corrected for multiple comparisons). Testing the overall USE – participant fit (t-test against zero), we find two of three dimensions to be significantly related to participant judgements (size: t_101_ = 9.66, p<0.001; approach: t_101_ = 3.13, p = 0.014; climate: t_101_ = 1.55, p = 0.75; corrected for multiple comparisons).

**3. Participant Variability: It would be interesting to know a bit more about the range of participant responses and how this might relate to the correlations observed with the DNNs. For instance, is the consistency of the correlation with DNNs robust even if individual response ranges vary? What were the range of responses? Did people use the whole range? Does that matter to the correlations with the DNNs?**

We agree that more detail on participant responses is needed. We therefore included additional visualizations in the Supplementary material. We show participant responses for each of the six dimensions in the Supplementary Fig. C (top panels, see our response to comment #2). Also, we included a new plot in the Supplementary material (Supp. Fig. B) where we quantified the range of responses (min, max) across dimensions for each participant. This reveals that the large majority of participants (95 %) responded within the -30 to +30 range of the visual rating scale (-50 to +50), suggesting only minor variations in the usage of the rating scale across participants. Concerning the influence of the rating distribution on the DNN correlation we argue that we are currently using Spearman correlations to compare the similarity matrices which uses a rank-metric that is invariant to the actual range of ratings. However, to make sure participant ratings do not differ in effects across the DNN similarity correlations, we computed Pearson correlations between the model to participant correlations and the subject-specific rating range (Supp. Fig. B). We did not find a significant relation between DNN – subject similarities and the range used for the similarity judgements. We included this result in the Supplement.

**Supp. Fig. B. Overview of participant ratings for Study I+II and relation to DNN similarities.** (A) Participants in Study I and II indicated similarity judgements on a visual analogue scale. We plotted histograms of minimum and maximum ratings per participant, suggesting only minor variations in the usage of the rating scale across participants (95% of participants within a range of -30 to 30). (B) Histogram of rating range (maximum rating – minimum rating) for all participants. (C) Finally, to test if the individual rating range would influence the correlation of participant ratings and DNN similarity matrices, we correlated the average DNN similarity for conv, fc and USE in each participant to the participants’ rating range (max – min) using Pearson correlation. We show that all three layer/model types do not relate to participant rating ranges (USE: *r* = 0.012, p = 0.90; fc: *r* = 0.12, p = 0.24; conv: *r* = -0.15, p = 0.13).

In our results section, we now report this:

*“To ensure that the range of rating responses used by participants did not influence the similarity between DNN-predicted and human behavioral judgements, we correlated participant-specific response ranges with the DNN matching, yielding non-significant relationships (Supplementary Fig. B in S1 Text).”* (p. 6, line 199-203)

**4. Visualizing Human-Model Correlations: The human-model correlation plots - why not show the individual dots? Are there too many? It would be nice to be able to see the distribution of the correlations, though not necessarily in all plots.**

We thank the reviewer for pointing this out. We now changed the barplot to a boxplot to be more informative and added participant-specific values (overlaid scatter points) to the layerwise DNN plot. We also now include this plot in the main manuscript in and added the results into the main manuscript.

**Fig. 1:** (F) Layerwise correlation analysis reveals higher model-human rating similarity for early to mid cDNN layers (conv) to perceptual ratings (positive values) and higher similarity of late cDNN layers (fc) and the semantic model to conceptual ratings (negative values).

*“First, we investigated if convolutional cDNNs layers (conv), fully connected layers (fc) and the semantic model (USE) would reflect human similarity judgements according to rating task instructions (Fig. 1F): We expected perceptual ratings to be related to visual cDNN and conceptual ratings to be related to semantic USE similarities. To this end, we analyzed layer-wise similarities using paired t-tests (one-sided), FDR corrected for multiple comparisons. Effect sizes are measured by Cohen’s d. Indeed, representational distances in cDNN layers conv1 (t_101_ = 1.86, p = 0.049, d = 0.18, perceptual: 0.024±0.1 (M±SD), conceptual: 0.001±0.08), conv3 (t_101_ = 2.58, p = 0.017, d = 0.25, perceptual: 0.074±0.1, conceptual: 0.038±0.09), conv4 (t_101_ = 2.69, p = 0.017, d = 0.26, perceptual: 0.07±0.09, conceptual: 0.03±0.08) and conv5 (t_101_ = 2.88, p = 0.017, d = 0.28, perceptual: 0.08±09, conceptual: 0.04±0.08) predicted perceptual ratings better than conceptual ratings, while the reverse was true for fully-connected layer 8 (t_101_ = -1.93, p = 0.049, d = 0.19, perceptual: 0.10±0.09, conceptual: 0.13±0.10) and USE distances (t_101_ = -2.15, p = 0.037, d = 0.21, perceptual:0.04±0.10, conceptual: 0.07±0.09).”* (p. 5, line 171-184)

For Supplementary Fig. F, we do not have participant ratings since this task consisted of binary ratings only (“choose the most similar image to the target image”). Therefore we cannot plot participant ratings here, but make this more clear now in the figure caption:

Supplementary Fig F Caption:
*“We computed similarities scores based on DNN model similarity matrices only since for Study III we only have the binary ratings which image was rated as more similar to the target.”*

**5. Grouping DNN Layers: the convolutional and fully connected layers were grouped - could the authors justify this (rather than looking at all layers) in methods or somewhere please.**

We thank the reviewer for the suggestion to further motivate our choice of averaging layers in the cDNN. In DNNs, it is often challenging and speculative to interpret the behavior and representational format of individual layers. While some layers clearly show the underlying processing features when visualized (i.e., object parts in convolutional layer 5 of an AlexNet), other layer visualization are hard to interpret since these layers are processing low-level features that are difficult to understand without additional context (for more details and an exemplary visualization of different layers see review by Heinen et al. 2023). Additionally, architectural similarities among the two layer types suggest similar representations in these layers. This shared representation can be visualized via cross-correlation matrices, that we included in the revised version of the manuscript in Fig.1E. Lastly, in this study we employ the DNNs as markers for perceptual and conceptual representational formats in behavioral measures. While on a neural level, layerwise correlations to brain activity might reveal interesting insights into neural representations for each region of interest along the ventral visual pathway, layerwise predictions to behavioral measures remain speculative.

Thus, to demonstrate and further motivate our choice to average, we added the following in the results section:

*“Based on these results, the architectural differences between convolutional layers and fully-connected layers, and the cross-correlation matrix (Fig. 1E), we computed layer averages for the cDNN (see Methods for more details) of conv and fc layers.“* (p. 5, line 185-187)

And we included a cross-correlation matrix to Fig.1(p. 5, line 171-184):

**Fig. 1:** (E) Layerwise and model cross-correlation reveals similarity clustering for convolutional layers, fully connected layers and distinct representations for the semantics model.

And added the cross-correlation to the Methods:

*“In addition, a cross-correlation of layers and models suggested high similarities between convolutional layers and between fully connected layers, while also demonstrating that the semantic model represents a metric distinct from the visual cDNN (Fig. 1E).“* (p. 29, line 921-924)

**6. LSQR: As a minor point, it might be interesting to briefly discuss potential ways to validate the LSQR sparse matrix regression, perhaps by predicting out of sample stimulus distances.**

We thank the reviewer for this comment. In the revised version of the manuscript, we have decided to address this point by including a quantitative (inferential) approach to test the validity of space reconstructions. Specifically, we validated the reconstruction approach by comparing the error from the least-squares solutions from the empirical data with shuffled responses in a permutation test. To do so, we repeated the least-squares estimation for each individual dimension 10.000 times, each time shuffling the order of the behavioral response vector of rated distances while keeping the design matrix M constant (i.e. same pairs in each iteration). From each iteration, we extracted the error of the model fit, built a distribution of errors in random permutations and finally ranked the empirical value in the surrogate distribution to obtain a p-value of the empirical model fit. This approach is now implemented in the revised version of the manuscript in Fig. 3C, the results and the method section:

**Fig. 3:** (C) Validation of LSQR space reconstruction by comparing the estimated LSQR error to the error obtained from models that predict randomly shuffled behavioral ratings.

*“To test the validity of space reconstructions, we performed a premutation test, shuffling the rating response vectors while keeping the design matrices M constant to build a surrogate distribution of randomized relationships and ranked the empirical error of the LSQR model fit in the surrogate distribution (see Methods for more details; Fig. 3C). Indeed, all reconstructed spaces reflect behavioral responses significantly better than chance (all p<0.001; Fig.3C).”* (p. 9, line 274-278)

*“To test if our reconstructed spaces indeed predict behavioral responses better than chance, we computed a random distribution of image x rating pairings based on 10.000 repeats and extracted the corresponding LSQR error estimate. We then plot the estimated error from the real ratings and the distribution of the shuffled random data (Fig. 3C).”* (p. 28, line 891-894)

Additionally, we now show the participant to global space correlations to demonstrate that the space reconstructions of the same space and the individual participant spaces are substantially consistent in Fig. 3D (see response to comment#1), a section in the results and in the methods:

*“Next, we investigated how each individual participant space would relate to the global space based on all participant ratings. We computed the participant to global space similarity using spearman rho correlations of the reconstructed global space similarity matrix and the reconstructed individual similarity matrix of each participant (Fig. 3D).* *We then tested if this participant to global space fits differed between spaces and format using a t test (perceptual vs. conceptual). Next, we tested if the variance of participant – global fits would differ between spaces by computing Levene tests for the participant – global correlations between the six spaces.”* (p. 28, line 895-902)

*“Indeed, all reconstructed spaces reflect behavioral responses significantly better than chance (all p<0.001; Fig.3C). Interestingly, when comparing how global space maps (i.e. reconstructed based on the entire sample) reflect maps from single participants, we find higher correlations for conceptual as compared to perceptual spaces (t_101_ = 17.40, p<0.001; Fig. 3D), while the variance of participants global matching was comparable across spaces (all Levene tests p>0.05).”* (p. 9, line 277-282)

We also added a short outlook on using out of sample concepts to predict their location and relations in abstract spaces (i.e., shortcuts in cognitive spaces) into the discussion:

“*It would also be interesting to see if the spaces in our study enable shortcuts by predicting relations for leave-out concepts*.” (p. 21, line 622-624)

**Overall, this is a high-quality manuscript. Addressing these minor points would likely enhance the clarity and impact of this work.**

# **Reviewer #2**

**The present manuscript, "Task-relevant representational spaces in human memory traces," reports on a study investigating how task demands during encoding, consolidation, and retrieval shape the structure and format of memory traces. Across a series of behavioral experiments, participants compared pairs of natural images (primarily animals) along either conceptual (e.g., size, climate) or perceptual (e.g., color, orientation) dimensions. These comparisons embedded the images into multidimensional "representational spaces," the structure of which was quantitatively modeled using deep neural networks (DNNs) to approximate both perceptual and semantic features.
A key finding is that distances between items in task-relevant representational spaces—those dimensions emphasized during encoding—predicted subsequent memory strength: items that were more distinct along these relevant dimensions were better remembered. In contrast, distances in task-irrelevant spaces showed no such predictive effect. Interestingly, conceptual encoding did not impair the ability to reject perceptually similar lures, suggesting that perceptual details remain accessible within the memory trace even when not emphasized during encoding.
The study also examined the role of consolidation through targeted memory reactivation (TMR) during sleep. Sounds previously paired with either conceptual or perceptual encoding were replayed during sleep, selectively enhancing memory for the corresponding representational format. However, strengthening conceptual representations through TMR appeared to reduce access to perceptual detail, as evidenced by impaired discrimination of similar lures.
Taken together, the results suggest that memory traces are flexible and encompass multiple coexisting representational formats, with their relative accessibility shaped by both encoding instructions and consolidation processes. This flexibility enables memory to support both specific (detail-rich) and generalized retrieval demands.
The manuscript addresses an important and timely topic. However, the methods section is currently difficult to follow due to the number of experimental variations and manipulations. A more structured and transparent presentation is recommended. In addition, some assumptions and analytic decisions require further justification. Below, I outline specific major and minor comments and suggestions for improvement.**

We thank the reviewer for the critical evaluation of our work. In the revised version of the manuscript, we have carefully addressed each point.

**Major Comments**

**1. Implementation of conceptual vs. perceptual dimensions:
It is not fully clear in how far size judgements are less ‚perceptual‘ than orientations of the animals. Given that the conceptual vs. perceptual manipulation is critical for the presented studies, the authors should clearly justify their choices for this manipulation.**

We agree with the reviewer that the choice of dimensions needs more clarification. For conceptual dimensions, we chose features that could be rated by only knowing the name of the concept (i.e., rating if you approach a shark or a turtle), independent from the physical features of the individual exemplars. For perceptual ratings we chose dimensions that would require seeing an actual image (i.e., the orientation of an animal). Concerning the size dimension, we instructed participants to rate the real-world size of the animals, not their size on the image. In addition, we chose images from THINGS that show the animal to be rated in focus and filling out the image, thus size could not be guessed by just looking at the images.

*“Deciding on the rating dimensions, we chose judgements that would either require an exemplar (i.e., image; Does an animal look straight at you or to the side for orientation) to actually judge its similarity for perceptual ratings, while for conceptual ratings, we chose judgements that would only require the animal name (i.e., comparing elephant and mouse for real-world size).“* (p. 24, line 736-743)

Further our DNN results support the notion that participants indeed processed different representational formats according to task-instructions. This is reflected in both response times and rated distances that could be predicted by conv, fc and USE model features. Computing dimension-wise DNN correlations were able to show that the size dimension ratings are predicted by higher-level and semantic DNN similarities and not by the visual layers, suggesting that participants indeed rated the size dimension based on the real-world size and not the size of that animal in the image.

**2. Use of DNNs as Ground Truth for Representational Formats:
In this study, DNNs are used as models to differentiate perceptual from conceptual processing. However, the introduction does not provide sufficient justification for (a) why DNNs should serve as a proxy for human processing, and (b) why the specific network implementations are appropriate models for distinguishing between perceptual and conceptual representations in human. Without this, the statement “Taken together, these results demonstrate that our rating task manipulated participants’ employment of task-relevant spaces” appears overstated. A more cautious interpretation would be that human similarity ratings show correlational associations with different DNN layers, in a manner consistent with theoretical assumptions about perceptual and conceptual processing hierarchies in DNNs. Here and throughout the manuscript the authors should opt for more descriptive interpretations in the result section and use more interpretative speculations only in the discussion section.**

We thank the Reviewer for this comment, which allows us to clarify our justification for using DNNs as a proxy for processing of perceptual and conceptual information in the human brain and, specifically, for using a DNN with this particular architecture. Nevertheless, we agree that a more cautious wording is more appropriate and have clarified throughout the manuscript that DNNs only provide correlational evidence for the employment of specific representational formats.

Regarding the utility of DNNs, we employed them as a proxy of the hierarchy of information processing from perceptual to conceptual dimensions because previous studies have shown that the representational structure of DNNs – and in particular of the DNN “AlexNet” employed in our study – shows a significant match with the representational structure in the human ventral visual stream ( (Güçlü und van Gerven 2015; Khaligh-Razavi und Kriegeskorte 2014); actually, it was even shown that *only* DNNs but not unsupervised models explain variance in mid-level inferior temporal cortex (Khaligh-Razavi und Kriegeskorte 2014). On the other hand, these models could only account for part of the variance in neural representations, most likely because at the implementational level most common DNNs differ strongly from information processing in the brain. This is now also explicitly stated:

*“ The processing hierarchy observed in convolutional deep neural networks (cDNNs) trained for object recognition provides a framework for understanding the representational formats (Gifford et al. 2022; Graumann et al. 2022; Clarke et al. 2018; Kietzmann et al. 2019; Kuzovkin et al. 2018; Allen et al. 2022; Güçlü und van Gerven 2015): previous studies have demonstrated that the representational structure of DNNs significantly corresponds to representations and processing steps along the ventral visual pathway (Güçlü und van Gerven 2015; Khaligh-Razavi und Kriegeskorte 2014).”* (p. 2, line 70-74)

Apart from the match with neural representations, several previous studies have shown that DNN representational similarities match with behavioral similarity judgements (Mur et al. 2010; Hebart et al. 2020). Regarding the specific architecture, we employed the DNN “AlexNet” for several reasons. First, it aims to model the hierarchy of processing from perceptual to conceptual information in the ventral visual stream (Güçlü und van Gerven 2015). Second, it is one of the most commonly used models and has been employed to capture representational structure during perception (Güçlü und van Gerven 2015; Khaligh-Razavi und Kriegeskorte 2014), short-term memory (Liu et al. 2020), long-term memory (Liu et al. 2021), and conceptual and perceptual memory (Davis et al. 2021). Finally, AlexNet pretrained on the ImageNet dataset with the objective to identify animal concepts is consistent with our task to compare animals on different conceptual dimensions – requiring participants to extract semantic knowledge about perceptually invariant animal concepts. Complementary to DNNs, which predictions are limited to the VVS (Bonnen et al. 2021), large language models capture purely semantic similarities and have been found to match human behavior and neural representations (Schrimpf et al. 2021; Caucheteux et al. 2022; Caucheteux und King 2022; Pereira et al. 2018).

This is also now explicitly stated in the introduction:

*“However, representations beyond the ventral visual pathway are not reflected in cDNN representations (Bonnen et al. 2021). Thus, as representational formats gets more complex and semantic, there is need for other models to reflect human brain responses and behavior, for example corpus-based models of deep natural language processing (dNLP) that use text rather than images as input (Lee und Chen 2022). Indeed, recent studies found that representations of these language models matches human neural and behavioral similarities during language processing and narrative tasks (Schrimpf et al. 2021; Caucheteux et al. 2022; Caucheteux und King 2022; Pereira et al. 2018; Lee und Chen 2022). Similarities between DNN and human representations are not limited to perception, but have been demonstrated similarity judgements (Mahner et al. 2025; Jozwik et al. 2017), short-term memory (Liu et al. 2020; Rau et al. 2025), long-term memory (Liu et al. 2021) and perceptual and conceptual memory (Davis et al. 2021). Thus, cDNNs and dNLPs provide different quantitative models of cognitive representations across various representational formats (Doerig et al. 2023; Heinen et al. 2023; Liu et al. 2021; Saxe et al. 2021).“* (p. 2-3, line 74-84)

We also rephrased our conclusion in the result section for the DNN and human rating relations:

*“Taken together, these results suggest that human similarity judgements of perceptual or conceptual format features can be predicted using similarities from visual and semantic DNNs, respectively.”* (p. 6, line 205-207)

And we included our motivation to use AlexNet in the Method section:

*“We chose AlexNet for mainly two reasons: First, previous studies have linked the representations of this network to human neural and behavioral similarities (Güçlü und van Gerven 2015; Liu et al. 2020; Liu et al. 2021; Rau et al. 2025), second while other studies make use of deeper cDNNs such as VGG-16 (Simonyan und Zisserman 2014), AlexNet consists of only 8 layers, which offers deeper insights into the “blackbox” of DNNs and higher levels of interpretability even of intermediate layers.”* (p. 29, line 906-911)

It should be mentioned that recent studies suggest that other model architectures such as recurrent cDNNs (Kietzmann et al. 2019) but also initial training weights can influence the relation between DNN similarities and the human brain and behavior. Testing these models on our task will be an interesting future direction and this point is now included in the discussion:

*“Future studies should also focus on testing additional DNNs and their relation to human similarity judgements since model architecture (such as recurrent connections between layers (Kietzmann et al. 2019)), and even starting weights can influence DNN representations (Mehrer et al. 2020).”* (p. 22, line 692-695)

**3. Recognition Memory Analyses:
Recognition memory outcomes should be reported in more detail. Confidence ratings should be presented separately for old and new items—and, if applicable, for different categories of new items (e.g., similar lures vs. novel foils). Furthermore, it would be valuable to determine whether recognition performance was above chance. One approach would be to dichotomize the confidence scale (e.g., using the midpoint as a neutral threshold) and classify responses into hits, misses, correct rejections, and false alarms. This would allow evaluation of recognition performance using standard signal detection theory metrics (cf. Macmillan & Creelman, 2004).**

We agree that more detailed information on recognition confidence and performance measures improves the description of behavioral responses in the memory tasks. Therefore, we extended the results section by reporting confidence of memory responses, separately for old exemplars, new exemplars (i.e., similar lures), and new concept images (Fig. 2A).

Further, we tested that discrimination performance, expressed as area under the curve and d prime, and included ROC plots that show the relationship between Hits and False alarms in bins of confidence across the response scale (for studies I + II: Fig 2B, study III: Fig. 4C).

**Fig. 2:** (A-B) (A) Confidence ratings for old images and lures (new exemplars and new concepts). Higher values indicate higher confidence for hits (old items) or correct rejections (new items). (B) ROC and AUC for Study I and II indicate successful discrimination between old images and lures.

**Fig. 4:** (C) Encoding task does not influence memory strength. However, we find better discrimination performance in the general memory test.

We added the linear mixed model and the ROC statistics to the Results and Methods:

*“**First, we tested if participants were able to discriminate between old images, new exemplars and new concepts during recognition using one-sample t-tests and receiver-operator characteristics (ROC). We find confidence for all three stimulus types to be significantly different from zero (old: t_46_ = 5.75, p<0.001 (SI), t_54_ = 3.87, p = 0.003 (SII); new exemplars: t_46_ = 7.96, p<0.001 (SI), t_54_ = 8.32, p<0.001 (SII); new concepts: t_46_ = 14.93, p<0.001 (SI), t_54_ = 16.84, p<0.001 (SII); Fig. 2A). Participants successfully discriminated old images against lures (area under the curve (AUC) new exemplars: t_46_ = 26.45, p<0.001 (SI); t_54_ = 15.60, p<0.001 (SII); AUC new concepts: t_46_ = 26.63, p<0.001 (SI); t_54_ = 22.62, p<0.001 (SII)) and were better at rejecting new concepts as compared to new exemplars (AUC SI: t_46_ = -15.28, p<0.001; SII: t_54_ = -14.45, p<0.001; Fig. 2B) with d-prime substantially above zero (old vs. new exemplars: d’ = (mean±sd) 0.72±0.26; t_46_ = 18.58, p<0.001 (SI), d’ = 0.63±0.28, t_54_ = 16.34, p<0.001 (SII); old vs. new concept: t_46_ = 20.03, p<0.001, d’ = 1.25±0.42, d’ = 1.23±0.46, t_54_ = 19.07, p<0.001 (SII)).****”*** (p. 8, line 237-249)

*“In addition, we found better discrimination of old images against lures (AUC old vs. new exemplars: t_27_ = 25.18, p<0.001; AUC old vs. new concepts: t_27_ = 24.03, p<0.001) and better discrimination between lures (new exemplars vs. new concepts) for general memory (AUC t_27_ = -9.52, p<0.001) compared to specific memory (AUC t_27_ = -29.01, p<0.001; Fig. 4C, right), while d-prime was substantially above zero (general memory: old vs. new exemplar: d’ = (mean±sd) 0.45±0.51, t_27_ = 4.75, p < 0.001; old vs. new concept: d’ = 1.87 ±0.66, t_27_ = 14.96, p < 0.001; specific memory: old vs. new exemplar d’ = 1.50±0.47, t_27_ = 16.88, p < 0.001; old vs. new concept: d’ = 2.21 ±0.54, t_27_ = 21.67, p < 0.001).”* (p. 12, line 353-360)

*“Lure discrimination performance*

*To test if participants successfully discriminated old images from lures, we computed receiver operator characteristics (ROC). To this end, we divided our continuous confidence rating scale into bins of 5 and tested whether the area under the curve (auc) across all confidence bins was above chance (0.5) using one-sample t-tests (Fig. 2B; Fig. 4C). In addition, we computed d’ tested if d’ was different from zero using one-sample t-tests.”* (p. 27, line 843-850)

**4. Use of the Term “Encoding Space”:
„We first analyzed whether the encoding space of an item (perceptual or conceptual) affected the memory strength of that item.“
The phrase “encoding space” implies an interpretive conclusion that may not be justified at the level of the manipulated variables. In the experiments, participants performed one of two encoding tasks (conceptual vs. perceptual). Whether these tasks give rise to separable “encoding spaces” is an inference based on the results, not a direct manipulation. This distinction should be reflected in the manuscript by using more neutral language during result reporting and reserving theoretical terms for the interpretation sections.**

We thank the reviewer for this comment and agree that the term “encoding space” may be overly speculative. We have rephrased respective sentences throughout the methods and results section to state “encoding task/s” instead of encoding space/s.

**4. Lack of Memory Advantage for Conceptual Encoding:
The finding that recognition memory did not differ between perceptual and conceptual encoding is surprising, given the well-established levels-of-processing framework (Craik & Lockhart, 1972), which posits that deeper (conceptual/semantic) processing enhances memory retention. Numerous studies have since demonstrated superior memory for semantically encoded material. The manuscript should address this discrepancy and provide possible explanations for the absence of a conceptual encoding advantage.**

We agree that this finding deserves more thorough evaluation. To address this point, we included an extended paragraph in the discussion section of the revised manuscript.

*“The levels of processing (LOP) theory posits that memory performance depends on the depth of processing during encoding, yielding better memory for conceptually (deep) as compared to perceptually (shallow) encoded stimuli. Contrary to this prediction, neither our study nor other investigations have found a consistent main effect of encoding format on later memory While unexpected, our results are in line with other studies finding no general effect of processing depth on subsequent memory (Delhaye und Bastin 2021; Bainbridge 2020). Several factors may explain this discrepancy. First, while many LOP studies predominantly use words as stimuli, images are less frequently employed (Ovalle-Fresa et al. 2021), and the extent to how task instructions engage differential processing depth of natural images remains unclear. Additionally, in our paradigm, image durations were relatively long (1.5 seconds) as compared to presentation durations in the order of only a few hundred milliseconds (Jimenez et al. 2020) and we can therefore not rule out that conceptual features were equally extracted during perceptual encoding conditions. Indeed, during object recognition, semantic category can be decoded after a few hundred milliseconds in inferior temporal cortex (DiCarlo et al. 2012) suggesting that automated extraction of conceptual features were possible in both encoding conditions. Interestingly, a recent study showed that image features, measured as reconstruction error of a cDNN, and not the orientation to specific features predicts later memory (Lin et al. 2024). Third, our paradigm involved pairwise comparisons of images, introducing a working memory component not typically present in classical LOP tasks, which rather present single items or multiple items simultaneously (Cermak und Craik 2014). Interestingly, research has shown that both perceptual and conceptual formats are retained during working memory maintenance periods after initial encoding (Liu et al. 2020), suggesting that both types of representational formats contribute to neural representations of individual images.*

*Importantly, the relational judgement of image pairs afforded participants to process and respond to stimulus relationships (i.e. rated distance), rather than focusing on individual item or artificial visual features (i.e., a circle around a concept for perceptual judgements instead of features of the concept itself) (Dubravac et al. 2025). Accordingly, encoding depth may be less concerned with the individual stimulus features as in classical LOP studies, but more likely depended on the relational structure in task-dependent representational spaces. Further, pairwise comparisons likely necessitate the extraction of high-level meaning to evaluate pairwise item relationships. While it could be shown that the attribution of meaning influences subsequent memory (Brady et al. 2019), this may contribute equally to judgements based on perceptual or conceptual representational formats.”* (p. 19-20, line 559-590)

**5. Clarification of TMR Design and Figure 5B:
I may have missed a critical detail in the description of the TMR manipulation—if so, I apologize for the misunderstanding. However, I find the logic behind Fig. 5B somewhat confusing. As I understand it, each encoding block (conceptual vs. perceptual) was paired with a distinct sound, and during sleep, only one of these sounds was replayed. If, for instance, the sound associated with conceptual encoding was played during sleep, then only conceptually encoded items could have been cued. Consequently, the uncued items in that condition would necessarily be perceptually encoded. Under this setup, the left panel of Fig. 5B effectively compares conceptual vs. perceptual items—given that cueing and encoding conditions are fully confounded—making it difficult to attribute observed effects to cueing rather than to the original encoding condition.
This issue becomes even more complex when comparing Fig. 5B with Fig. 1G. Fig. 1G presents memory performance by encoding condition and suggests that conceptual encoding results in lower memory strength (with values below 5). In contrast, Fig. 5B shows memory scores for cued conceptual items around 8. Moreover, based on Fig. 1G, perceptual encoding appears to lead to better memory performance than conceptual encoding. These discrepancies are difficult to reconcile and raise questions about the internal consistency of the findings. In my view, a clearer and more transparent reporting of the memory results—especially to facilitate comparisons across studies within the manuscript—is essential.**

We apologize for the lack of clarity in our descriptions. Indeed, in one given participants, either only the perceptually encoded items or only the conceptually items were cued, and comparing them would be confounded. But actually, we compared conceptually encoded items in participants where these items were cued vs. conceptually encoded items in other participants where these items were not cued. The same was done for perceptually encoded items. We clarified this point in the revised version of the manuscript where we now write:

*“We compared conceptually encoded items in participants where these items were cued vs. conceptually encoded items in other participants where these items were not cued and vice versa for perceptually encoded items resulting in a between design.”* (p. 16, line 466-469)

We also removed the indeed misleading lines between conditions in Fig. 6B-C (former Fig. 5B-C):

**Fig. 6:** (B-C) Cueing of conceptual but not of perceptual formats increases memory strength. Cueing of perceptual formats improves and cueing of conceptual formats impairs correct rejection of new exemplars.

Further we added the term between-subject design to where we describe Study II:

*“We employed targeted memory reactivation (TMR) in a between-subject design and tested whether cueing with a sound that was presented during either perceptual or conceptual encoding selectively enhanced the corresponding representational format (Figure 6A).”* (p. 16, line 462-465)

We also added the following statement to the limitations part in the Discussion:

*“In addition, testing our sleep effects within the same participant would offer insights into interindividual differences in consolidation during sleep.”* (p. 22, line 683-685)

Regarding the apparent discrepancies of confidence values between Fig. 6 (former Fig. 5) and Fig. 2 (former figure 1) we agree with the reviewer that it was not clear which trials went into the analysis. We only use uncued trials from the sleep study, since we aim to investigate the effect of encoding condition (without the effect of cueing). We also want to make it comparable to the effects of Study I, where no cueing happened. Thus, for this analysis, we only included the uncued items for Study II. To make this clear, we now exactly state which trials were used:

*“Using a linear mixed model of trial-wise memory strength with “encoding task” as a predictor and participant-specific intercepts as random effects, we did not find effects for more accessible memory traces following conceptual vs. perceptual judgements (Study I: z = -0.36, χ^2^_(1)_ = 0.42, p = 0.516; Study II: z = 0.15, χ^2^_(1)_ = 0.02, p = 0.874; for Study II only uncued items were included in the analysis; likelihood ratio test; Fig. 2C).”* (p. 8-9, line 254-255)

**Minor Comments**

**6. Figure 4B:
The y-axis should begin at zero to improve interpretability and avoid misleading visual effects.**

We have changed the y-axis in Figure 5 (former Fig. 4) accordingly.

**7. Confidence Scale:
The confidence rating scale is insufficiently described in the methods and figures. Please provide the numeric range and explain how it maps onto qualitative confidence judgments (e.g., “sure new” to “sure old”).**

We agree with the reviewer that the description of confidence was not clear and inconsistent across the manuscript. Therefore, we added a description of the original scale values and how we rescaled them for plotting purposes. Since the original confidence scale had a range from -50 to 50, we decided to rescale it to 0 to 50 so that greater values would indicate higher confidence for both old images and lures. We now report this scale and its interpretation of values in

*“Participants rated similarities of the last presented image in relation to the first image on a visual rating scale (-50 to 50; Ratings of zero indicating the images score the same on the rated dimension i.e., polar bear and walrus both live in the same cold climate).“* (p. 24, line 736-739)

*“Afterwards, participants indicated their confidence of having seen the image before on a continuous rating scale (-50 to 50) from “sure old” (-50) to “sure new” (50). All 336 old images, 168 new exemplars, and 168 new concepts were shown in a random order. For analysis, we rescaled the confidence ratings to a range of 0 to 50 so that higher values would reflect higher confidence for both the old images and the lures.”* (p. 25, line 791-796)

**9. Consistent Axis Scaling Across Figures:
Figures presenting the same metric (e.g., memory strength, confidence ratings) should use consistent axis ranges across the manuscript to facilitate direct visual comparison.**

We gladly followed this suggestion and scaled the axis ranges to be consistent for the same outcome measure (i.e., same scaling for same parameters for study I and II, and for study III).

**10. Inclusion of Response Time Results:
The reported finding that similarity judgments were faster when the distance between items along relevant dimensions was larger (Supplementary Fig. 2A) is important and should be included in the main text.**

We gladly followed the suggestion of the reviewer and moved the reaction time results formerly presented in Supplementary Fig. 2A into the main manuscript, now presented in Fig. 3E and the main results:

**Fig. 3:** (E) Cueing of conceptual but not of perceptual formats increases memory strength. Cueing of perceptual formats improves and cueing of conceptual formats impairs correct rejection of new exemplars.

*“First we ensured that rated distances were behaviorally relevant during encoding. Indeed, responses to images were given faster when their distances on relevant dimensions were larger (Study I: z = -11.28, χ2(1) = 126.69, p < 0.001; Study II: z = -6.67, χ2(1) = 44.49, p < 0.001; Fig. 3E; see Supplementary Fig. D in S1 Text* *for more detail on the reaction times analyses). Second, we investigated how the rated distances influenced subsequent memory.”* (p. 10, line 283-287)

References

Allen, Emily J.; St-Yves, Ghislain; Wu, Yihan; Breedlove, Jesse L.; Prince, Jacob S.; Dowdle, Logan T. et al. (2022): A massive 7T fMRI dataset to bridge cognitive neuroscience and artificial intelligence. In: *Nature neuroscience* 25 (1), S. 116–126. DOI: 10.1038/s41593-021-00962-x.

Bainbridge, Wilma A. (2020): The resiliency of image memorability: A predictor of memory separate from attention and priming. In: *Neuropsychologia* 141, S. 107408. DOI: 10.1016/j.neuropsychologia.2020.107408.

Bellmund, Jacob L. S.; Gärdenfors, Peter; Moser, Edvard I.; Doeller, Christian F. (2018): Navigating cognition: Spatial codes for human thinking. In: *Science (New York, N.Y.)* 362 (6415). DOI: 10.1126/science.aat6766.

Bonnen, Tyler; Yamins, Daniel L. K.; Wagner, Anthony D. (2021): When the ventral visual stream is not enough: A deep learning account of medial temporal lobe involvement in perception. In: *Neuron* 109 (17), 2755-2766.e6. DOI: 10.1016/j.neuron.2021.06.018.

Brady, Timothy F.; Alvarez, George A.; Störmer, Viola S. (2019): The Role of Meaning in Visual Memory: Face-Selective Brain Activity Predicts Memory for Ambiguous Face Stimuli. In: *The Journal of neuroscience : the official journal of the Society for Neuroscience* 39 (6), S. 1100–1108. DOI: 10.1523/JNEUROSCI.1693-18.2018.

Caucheteux, Charlotte; Gramfort, Alexandre; King, Jean-Rémi (2022): Deep language algorithms predict semantic comprehension from brain activity. In: *Scientific reports* 12 (1), S. 16327. DOI: 10.1038/s41598-022-20460-9.

Caucheteux, Charlotte; King, Jean-Rémi (2022): Brains and algorithms partially converge in natural language processing. In: *Communications biology* 5 (1), S. 134. DOI: 10.1038/s42003-022-03036-1.

Cermak, Laird S.; Craik, Fergus I.M. (2014): Levels of Processing in Human Memory (PLE: Memory): Psychology Press.

Clarke, Alex; Devereux, Barry J.; Tyler, Lorraine K. (2018): Oscillatory Dynamics of Perceptual to Conceptual Transformations in the Ventral Visual Pathway. In: *Journal of cognitive neuroscience* 30 (11), S. 1590–1605. DOI: 10.1162/jocn_a_01325.

Davis, Simon W.; Geib, Benjamin R.; Wing, Erik A.; Wang, Wei-Chun; Hovhannisyan, Mariam; Monge, Zachary A.; Cabeza, Roberto (2021): Visual and Semantic Representations Predict Subsequent Memory in Perceptual and Conceptual Memory Tests. In: *Cerebral cortex (New York, N.Y. : 1991)* 31 (2), S. 974–992. DOI: 10.1093/cercor/bhaa269.

Delhaye, Emma; Bastin, Christine (2021): Semantic and perceptual encoding lead to decreased fine mnemonic discrimination following multiple presentations. In: *Memory (Hove, England)* 29 (1), S. 141–145. DOI: 10.1080/09658211.2020.1849309.

DiCarlo, James J.; Zoccolan, Davide; Rust, Nicole C. (2012): How Does the Brain Solve Visual Object Recognition? In: *Neuron* 73 (3), S. 415–434. DOI: 10.1016/j.neuron.2012.01.010.

Doerig, Adrien; Sommers, Rowan P.; Seeliger, Katja; Richards, Blake; Ismael, Jenann; Lindsay, Grace W. et al. (2023): The neuroconnectionist research programme. In: *Nature reviews. Neuroscience* 24 (7), S. 431–450. DOI: 10.1038/s41583-023-00705-w.

Dubravac, Mirela; Sachdeva, Chhavi; Rothen, Nicolas (2025): Levels of Processing Effects on Memory for Color-Object Associations. In: *Journal of cognition* 8 (1), S. 25. DOI: 10.5334/joc.437.

Garvert, Mona M.; Dolan, Raymond J.; Behrens, Timothy Ej (2017): A map of abstract relational knowledge in the human hippocampal-entorhinal cortex. In: *eLife* 6. DOI: 10.7554/eLife.17086.

Gifford, Alessandro T.; Dwivedi, Kshitij; Roig, Gemma; Cichy, Radoslaw M. (2022): A large and rich EEG dataset for modeling human visual object recognition. In: *NeuroImage* 264, S. 119754. DOI: 10.1016/j.neuroimage.2022.119754.

Graumann, Monika; Ciuffi, Caterina; Dwivedi, Kshitij; Roig, Gemma; Cichy, Radoslaw M. (2022): The spatiotemporal neural dynamics of object location representations in the human brain. In: *Nature human behaviour* 6 (6), S. 796–811. DOI: 10.1038/s41562-022-01302-0.

Güçlü, Umut; van Gerven, Marcel A. J. (2015): Deep Neural Networks Reveal a Gradient in the Complexity of Neural Representations across the Ventral Stream. In: *The Journal of neuroscience : the official journal of the Society for Neuroscience* 35 (27), S. 10005–10014. DOI: 10.1523/JNEUROSCI.5023-14.2015.

Hebart, Martin N.; Zheng, Charles Y.; Pereira, Francisco; Baker, Chris I. (2020): Revealing the multidimensional mental representations of natural objects underlying human similarity judgements. In: *Nature human behaviour* 4 (11), S. 1173–1185. DOI: 10.1038/s41562-020-00951-3.

Heinen, Rebekka; Bierbrauer, Anne; Wolf, Oliver T.; Axmacher, Nikolai (2023): Representational formats of human memory traces. In: *Brain structure & function*. DOI: 10.1007/s00429-023-02636-9.

Jimenez, Mikel; Hinojosa, José Antonio; Montoro, Pedro R. (2020): Visual awareness and the levels of processing hypothesis: A critical review. In: *Consciousness and cognition* 85, S. 103022. DOI: 10.1016/j.concog.2020.103022.

Jozwik, Kamila M.; Kriegeskorte, Nikolaus; Storrs, Katherine R.; Mur, Marieke (2017): Deep Convolutional Neural Networks Outperform Feature-Based But Not Categorical Models in Explaining Object Similarity Judgments. In: *Frontiers in psychology* 8, S. 1726. DOI: 10.3389/fpsyg.2017.01726.

Kawakita, Genji; Zeleznikow-Johnston, Ariel; Takeda, Ken; Tsuchiya, Naotsugu; Oizumi, Masafumi (2025): Is my "red" your "red"?: Evaluating structural correspondences between color similarity judgments using unsupervised alignment. In: *iScience* 28 (3), S. 112029. DOI: 10.1016/j.isci.2025.112029.

Khaligh-Razavi, Seyed-Mahdi; Kriegeskorte, Nikolaus (2014): Deep supervised, but not unsupervised, models may explain IT cortical representation. In: *PLoS computational biology* 10 (11), e1003915. DOI: 10.1371/journal.pcbi.1003915.

Kietzmann, Tim C.; Spoerer, Courtney J.; Sörensen, Lynn K. A.; Cichy, Radoslaw M.; Hauk, Olaf; Kriegeskorte, Nikolaus (2019): Recurrence is required to capture the representational dynamics of the human visual system. In: *Proceedings of the National Academy of Sciences of the United States of America* 116 (43), S. 21854–21863. DOI: 10.1073/pnas.1905544116.

Kuzovkin, Ilya; Vicente, Raul; Petton, Mathilde; Lachaux, Jean-Philippe; Baciu, Monica; Kahane, Philippe et al. (2018): Activations of deep convolutional neural networks are aligned with gamma band activity of human visual cortex. In: *Communications biology* 1, S. 107. DOI: 10.1038/s42003-018-0110-y.

Lee, Hongmi; Chen, Janice (2022): Predicting memory from the network structure of naturalistic events. In: *Nature communications* 13 (1), S. 4235. DOI: 10.1038/s41467-022-31965-2.

Lin, Qi; Li, Zifan; Lafferty, John; Yildirim, Ilker (2024): Images with harder-to-reconstruct visual representations leave stronger memory traces. In: *Nature human behaviour* 8 (7), S. 1309–1320. DOI: 10.1038/s41562-024-01870-3.

Liu, Jing; Zhang, Hui; Yu, Tao; Ni, Duanyu; Ren, Liankun; Yang, Qinhao et al. (2020): Stable maintenance of multiple representational formats in human visual short-term memory. In: *Proceedings of the National Academy of Sciences of the United States of America* 117 (51), S. 32329–32339. DOI: 10.1073/pnas.2006752117.

Liu, Jing; Zhang, Hui; Yu, Tao; Ren, Liankun; Ni, Duanyu; Yang, Qinhao et al. (2021): Transformative neural representations support long-term episodic memory. In: *Science advances* 7 (41), eabg9715. DOI: 10.1126/sciadv.abg9715.

Mahner, Florian P.; Muttenthaler, Lukas; Güçlü, Umut; Hebart, Martin N. (2025): Dimensions underlying the representational alignment of deep neural networks with humans. In: *Nature machine intelligence* 7 (6), S. 848–859. DOI: 10.1038/s42256-025-01041-7.

Mehrer, Johannes; Spoerer, Courtney J.; Kriegeskorte, Nikolaus; Kietzmann, Tim C. (2020): Individual differences among deep neural network models. In: *Nature communications* 11 (1), S. 5725. DOI: 10.1038/s41467-020-19632-w.

Mur, M.; Meys, M.; Bodurka, J.; Bandettini, P.; Kriegeskorte, N. (2010): Relating neural object representations to perceptual judgments with representational similarity analysis. In: *Journal of Vision* 9 (8), S. 780. DOI: 10.1167/9.8.780.

Ovalle-Fresa, Rebecca; Uslu, Arif Sinan; Rothen, Nicolas (2021): Levels of Processing Affect Perceptual Features in Visual Associative Memory. In: *Psychological science* 32 (2), S. 267–279. DOI: 10.1177/0956797620965519.

Pereira, Francisco; Lou, Bin; Pritchett, Brianna; Ritter, Samuel; Gershman, Samuel J.; Kanwisher, Nancy et al. (2018): Toward a universal decoder of linguistic meaning from brain activation. In: *Nature communications* 9 (1), S. 963. DOI: 10.1038/s41467-018-03068-4.

Rau, Elias M. B.; Fellner, Marie-Christin; Heinen, Rebekka; Zhang, Hui; Yin, Qin; Vahidi, Parisa et al. (2025): Reinstatement and transformation of memory traces for recognition. In: *Science advances* 11 (8), eadp9336. DOI: 10.1126/sciadv.adp9336.

Saxe, Andrew; Nelli, Stephanie; Summerfield, Christopher (2021): If deep learning is the answer, what is the question? In: *Nature reviews. Neuroscience* 22 (1), S. 55–67. DOI: 10.1038/s41583-020-00395-8.

Schrimpf, Martin; Blank, Idan Asher; Tuckute, Greta; Kauf, Carina; Hosseini, Eghbal A.; Kanwisher, Nancy et al. (2021): The neural architecture of language: Integrative modeling converges on predictive processing. In: *Proceedings of the National Academy of Sciences of the United States of America* 118 (45). DOI: 10.1073/pnas.2105646118.

Simonyan, Karen; Zisserman, Andrew (2014): Very Deep Convolutional Networks for Large-Scale Image Recognition.
